# Discovery of Lipid Peroxidation Inhibitors from *Bacopa* Species Prioritized through Multivariate Data Analysis and Multi-Informative Molecular Networking

**DOI:** 10.3390/molecules24162989

**Published:** 2019-08-17

**Authors:** Tongchai Saesong, Pierre-Marie Allard, Emerson Ferreira Queiroz, Laurence Marcourt, Nitra Nuengchamnong, Prapapan Temkitthawon, Nantaka Khorana, Jean-Luc Wolfender, Kornkanok Ingkaninan

**Affiliations:** 1 Department of Pharmaceutical Chemistry and Pharmacognosy, Faculty of Pharmaceutical Sciences and Center of Excellence for Innovation in Chemistry, Naresuan University, Phitsanulok 65000, Thailand; 2 School of Pharmaceutical Sciences, EPGL, University of Geneva, University of Lausanne, CMU Rue Michel Servet 1, 1211 Geneva 4, Switzerland; 3 Science Lab Center, Faculty of Science, Naresuan University, Phitsanulok 65000, Thailand; 4 Division of Pharmaceutical Sciences, School of Pharmaceutical Sciences, University of Phayao, Phayao 56000, Thailand

**Keywords:** metabolomics, multivariate data analysis, molecular network, *Bacopa monnieri*, LC-MS

## Abstract

A major goal in the discovery of bioactive natural products is to rapidly identify active compound(s) and dereplicate known molecules from complex biological extracts. The conventional bioassay-guided fractionation process can be time consuming and often requires multi-step procedures. Herein, we apply a metabolomic strategy merging multivariate data analysis and multi-informative molecular maps to rapidly prioritize bioactive molecules directly from crude plant extracts. The strategy was applied to 59 extracts of three *Bacopa* species (*B. monnieri*, *B. caroliniana* and *B. floribunda*), which were profiled by UHPLC-HRMS^2^ and screened for anti-lipid peroxidation activity. Using this approach, six lipid peroxidation inhibitors **1**–**6** of three *Bacopa* spp. were discovered, three of them being new compounds: monnieraside IV (**4**), monnieraside V (**5**) and monnieraside VI (**6**). The results demonstrate that this combined approach could efficiently guide the discovery of new bioactive natural products. Furthermore, the approach allowed to evidence that main semi-quantitative changes in composition linked to the anti-lipid peroxidation activity were also correlated to seasonal effects notably for *B. monnieri*.

## 1. Introduction

Natural products (NPs) play an important role as a source of various pharmaceuticals and biologically active substances. However, the discovery of new bioactive NPs is challenging because of the inherent complex composition of crude natural extracts. Such extracts contain hundreds, if not thousands, of chemical constituents and the purification and identification of bioactive NPs by conventional methods is a time consuming multi-step procedure. Moreover, bioactive substances can be lost during purification and effort can be wasted in the unnecessary re-isolation of known NPs. Therefore, it is important to pinpoint bioactive candidates and recognize known metabolites (dereplication) early in the purification process in order to avoid the redundant isolation of known molecules [1,2].

Recently, metabolomics combined with multivariate data analysis (MVA) has proven to be an efficient tool to predict bioactive constituents in NP research [3,4,5,6,7]. Metabolomics aims at providing comprehensive qualitative and quantitative analysis of the whole set of metabolites (metabolome) present in a complex biological sample [8,9]. The most used analytical techniques in metabolomics are nuclear magnetic resonance (NMR) and mass spectrometry (MS) [10]. Generally metabolite profiling of natural extracts is achieved via high resolution ultra-high performance liquid chromatography (UHPLC), coupled to high resolution tandem mass spectrometry (HRMS^2^), which provides molecular formula and fragmentation information on most NPs in extracts in an untargeted manner [11]. Unsupervised or supervised multivariate data analysis such as principal components analysis (PCA) or orthogonal partial least squares (OPLS) are then needed to mine such data and highlight biomarkers. Alternative strategies have been developed to explore LC-HRMS^2^ metabolite profiling datasets with the aim of highlighting structural similarities between analytes and efficiently identify new compounds with potential therapeutic interest. Molecular network analysis (MN) [12,13] is a computer-based approach allowing the organization of fragmentation spectra from MS-based metabolomics experiments in order to dereplicate and eventually prioritize natural products of interest [14,15,16]. MN is generated based on the similarities of fragmentation patterns and, thus, indirectly allows the grouping of analytes with closely related structures. Networks can be built using the Global Natural Product Social Molecular Networking (GNPS) platform [17] or software such as Metgem or MS-Dial [18,19]. 

*Bacopa* is a genus of aquatic plants belonging to the Plantaginaceae family. Three species occur in Thailand: *B. monnieri*, *B. caroliniana* and *B. floribunda* [20]. Among them, only *B. monnieri* (Brahmi) has been reported as a herbal medicine in Ayurvedic medicine for learning and memory improvement [21]. The safety and efficacy of Brahmi extracts in animal models [22,23] and in clinical trials [24,25,26,27,28] have been proven and support its traditional uses. Intake of Brahmi has been reported to exert undesirable effects on the gastrointestinal tract, such as nausea, increased stool frequency and abdominal cramps [25,29], which might be explained by a cholinergic effect [30]. In addition, severe liver toxicity has been detected in women taking Brahmi products for vitiligo disease. Nevertheless, their liver function returned to normal after discontinuation of products’ usage [31]. Other reports however indicated that Brahmi possessed hepatoprotective activity [32,33]. Notwithstanding such adverse effects and considering the positive effects of the plant in relation with cognition improvements, further investigations are still worth to identify bioactive principles.

The compounds responsible for the memory enhancing effects of Brahmi have been reported to be triterpenoid saponins i.e., bacoside A_3_, bacopaside I, bacopaside II, bacopasaponin C and bacopaside X [34,35]. They are considered as markers of Brahmi [36,37,38,39,40,41], and their level is assessed for quality control purposes. Usually, the level of plant specialized metabolites is highly variable according to environmental factors. In Brahmi, the levels of such markers were found to vary significantly depending on the part of used (leaves, stems, shoots etc.), collection area and season [42,43,44,45].

Moreover, this plant also contains other classes of NPs such as sterols [46], flavonoids [47] and phenylethanoids [48,49] that may play roles in the pharmacological activities of the plant. It has also been reported that part of the neuroprotective effects of Brahmi appeared to result from its antioxidant activities that suppress neuronal oxidative stress. Brahmi has been found to inhibit the lipid peroxidation reaction of brain homogenate in a dose-dependent manner [50]. 

In this study, we aimed at searching for compounds that could be involved in the memory improvement activity of Brahmi through lipid peroxidation inhibitory activity. In addition, the anti-lipid peroxidation activity of two other *Bacopa* species has been investigated. To achieve these goals, a metabolomic strategy combining multivariate data analysis (MVA) and bioactivity informed molecular maps [14] was used as a guide to highlight bioactive constituents early in the phytochemical study process and directly target their isolation.

## 2. Results and Discussion

**Figure 1:**
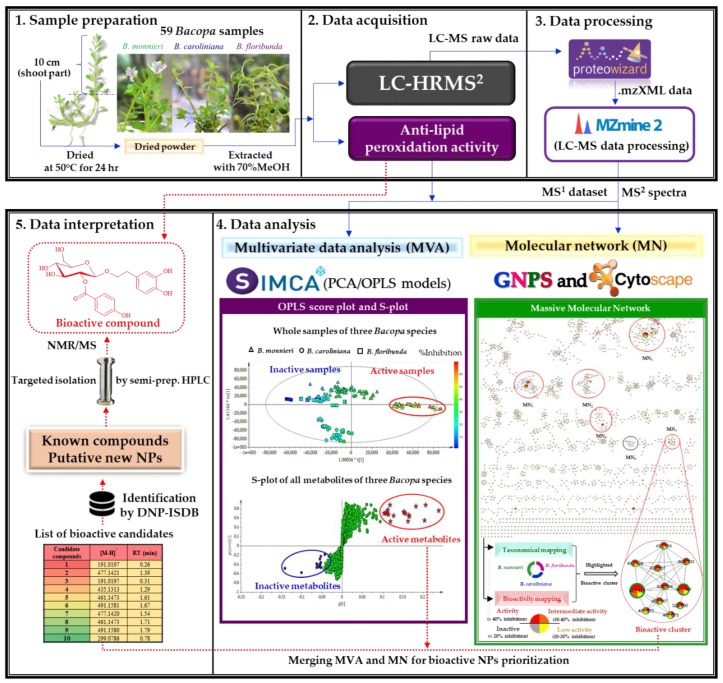
Schematic diagram of lipid peroxidation inhibitor discovery from LC-HRMS^2^ analyses of 59 *Bacopa* extracts combining metabolomics MVA and multi-informative MN.

Fifty-nine extracts of three *Bacopa* species from different regions of Thailand and harvested at various seasons [summer (March to June), rainy season (July to October) and winter (November to February)] were collected for this study. All extracts were profiled by UHPLC-HRMS^2^ to generate data that could be used to monitor metabolite profile variations across the whole dataset and provide high quality data dependent MS^2^ spectra for annotation. In parallel, all of the extracts were screened for their anti-lipid peroxidation activity. Variations in the profiles were then linked to bioactivity modulation through MVA in order to highlight possible bioactive metabolites. In addition, the MS^2^ dataset was organized using the GNPS platform to generate a MN, which was visualized using Cytoscape software. The bioactivity and taxonomy of plant extracts were mapped on the MN in order to pinpoint cluster(s) of potentially bioactive metabolite(s). The lists of prioritized candidates from MVA and MN were finally compared and the common metabolites were then selected as bioactive candidates. They were annotated based on their MS^2^ spectra compared with experimental or in silico MS/MS database (GNPS libraries and DNP–ISDB). Both known and possibly novel compounds were isolated to establish their bioactivities and their structures were unambiguously determined by NMR. A summary of the prioritization workflow is presented in Figure 1.

### 2.1. Lipid Peroxidation Inhibitory Activity Evaluation of the Extracts

**Figure 2:**
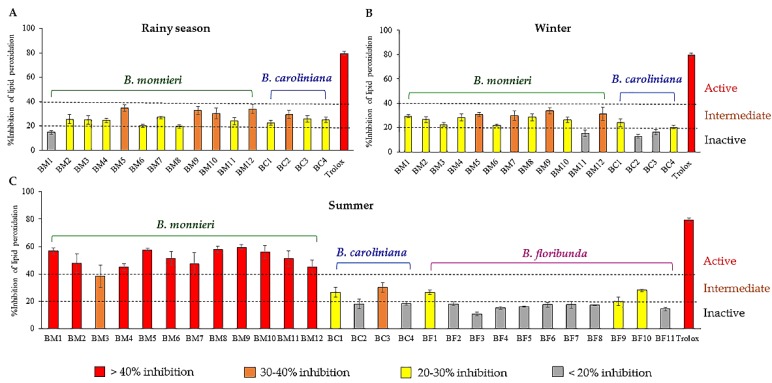
Anti-lipid peroxidation activities of three *Bacopa* species i.e., *B. monnieri* (BM), *B. caroliniana* (BC), and *B. floribunda* plant (BF) collected from different regions in the (**A**) rainy, (**B**) winter and (**C**) summer seasons. Samples giving >40% inhibition were considered active, 30‒40% inhibition was intermediate activity, 20‒30% inhibition was low activity, and those exhibiting <20% inhibition were considered inactive. Trolox (100 µg/mL) was used as a positive control.

The fifty-nine extracts of three *Bacopa* species collected from different regions of Thailand in rainy season, winter and summer were submitted to a thiobarbituric acid reactive substances (TBAR) assay. A significant variation of lipid peroxidation inhibitory activities between groups of related samples was observed (Figure 2A–C). In particular, *B. monnieri* harvested in summer (Figure 2C) exhibited stronger inhibitory effects (around 2-fold) than *B. monnieri* collected in other seasons or than other *Bacopa* species.

### 2.2. Potential Bioactive Metabolites Prioritized through Multivariate Statistical Analysis and Molecular Networking 

#### 2.2.1. Organization and Pre–Treatment of the Metabolite Profiling Data

All extracts that were screened for bioactivity were profiled by UHPLC-HRMS^2^ using a generic gradient in negative ionization (NI) mode to provide MS^1^ and MS^2^ data of all metabolites in the *Bacopa* samples. The NI mode was used because it provided far more molecular ion features than the positive ionization (PI) mode for the samples considered. This was in good agreement with the rich polyphenolic content of *Bacopa* species.

After profiling, the LC–HRMS^2^ data was treated by MZmine [51] for mass detection, chromatogram building, deconvolution, isotopic peak grouping, alignment and gap filling. This resulted in a peaklist of 6082 features which was further filtered to a peaklist of 4191 features having associated MS^2^ spectra. This peaklist of 4191 features was exported as input for the MVA (MS^1^ data only) and for MN generation (MS^1^ and MS^2^ data). These data were correlated to the extract’s bioactivity results in order to highlight bioactive compounds responsible for anti–lipid peroxidation in *Bacopa* species.

#### 2.2.2. Multivariate Data Analysis

**Figure 3:**
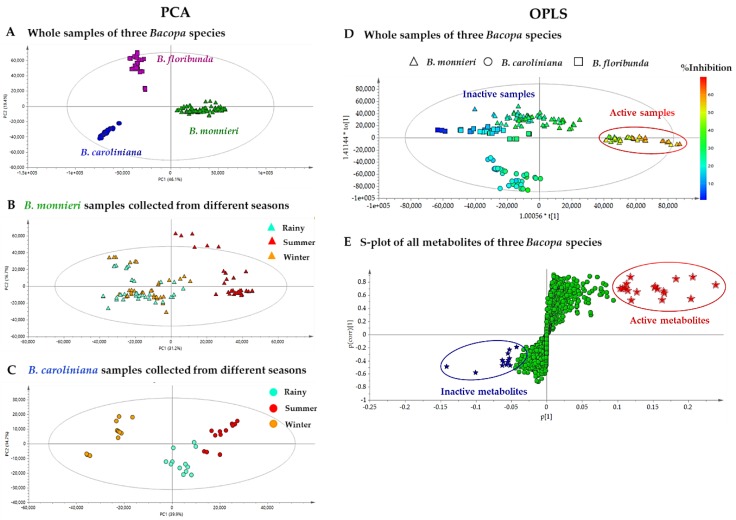
(**A**) PCA score plot based on chemical profiles of 59 extracts of three *Bacopa* species. (**B**,**C**) PCA score plots based on chemical profiles of 36 extracts of *B. monnieri* and 12 extracts of *B. caroliniana*, respectively from different sources in three seasonal collections. (**D**) OPLS score plot based on the chemical profiles and anti-lipid peroxidation activity of all *Bacopa* extracts. (**E**). S-plot presenting nineteen candidate active features with high p[1] values (red filled star) and inactive features with low p[1] value (blue filled star).

As a preliminary step, the whole MS^1^ dataset (consisting of *m/z* values, retention times (RT), and intensities) was analyzed by principal component analysis (PCA) to investigate the differences of metabolite profiles of three *Bacopa* species and the effects on quality of regional cultivation and seasonal harvesting of BM and BC samples. The PCA scatter plot (normalized by Pareto-scaling) is presented in Figure 3A. It showed obvious discrimination among *B. monnieri* (BM), *B. caroliniana* (BC) and *B. floribunda* (BF), exhibiting 65.50% of the total variance in the dataset (46.10% of the variance for PC1 and 19.40% for PC2). This plot exhibited obvious inter–species variations, while intra–species variation of BM and BC samples could only be observed in the PCA plots generated from the individual datasets of BM and BC (Figure 3B,C). Interestingly, the PCA plot of the BM dataset showed a clear separation between BM samples collected in summer versus those harvested in the rainy season and winter (Figure 3B). For BC, the samples were clustered into three groups, according to the harvesting season (Figure 3C). These results demonstrated that the metabolite profiles of BM and BC in different seasons were different and could thus impact the bioactivity of these samples. Therefore, notably for BM, which is used as a food supplement, the harvesting season clearly needs to be taken in consideration to favor the sought-after bioactivity. On the other hand, the PCA plots indicated that the sample composition did not seem to be affected by the region of provenance. 

In order to correlate the variations observed in bioactivities with the metabolite profiles of all extracts, the data was analyzed by a supervised method (OPLS), which is a regression extension of PCA allowing maximization of the separation between groups of observations and pinpointing of variables contributing to the separation. The peaklist consisting of *m/z* values, RT, and intensities was used as X variables (similarly to what had been done for PCA) and the %inhibitions of lipid peroxidation were used as the Y variables. A significant separation between the active and the inactive groups was observed, as shown in the OPLS score plot (Figure 3D), where a reddish color represents a high %lipid peroxidation inhibition. As expected from the initial screening results (see Figure 2), all samples exhibiting an activity higher than 40% were grouped (BM samples collected in summer). From the S-plot of all metabolites in the *Bacopa* samples (Figure 3E), 19 features at the upper right corner (highlighted with red stars) were identified as the most discriminating features between the active and non-active samples, and were thus potentially responsible for the observed anti-lipid peroxidation effects. In contrast, the features at the lower left corner corresponded to metabolites that were likely non-actives. These nineteen features with high p[1] values were thus ranked as putative bioactive features (Table 1). Using the *m/z* and RT of each feature (labeled **F**–**n°** in the Table 1), we found that seventeen features corresponded to unique compounds and that two other features, **F2** and **F8** were adduct and dimer forms of **F18** and **F3**, respectively. Therefore, seventeen bioactive candidates were prioritized from the S-plot. In parallel to this MVA treatment, the same dataset was explored using the multi-informative MN strategy.

#### 2.2.3. Multi-Informational Molecular Map

**Figure 4:**
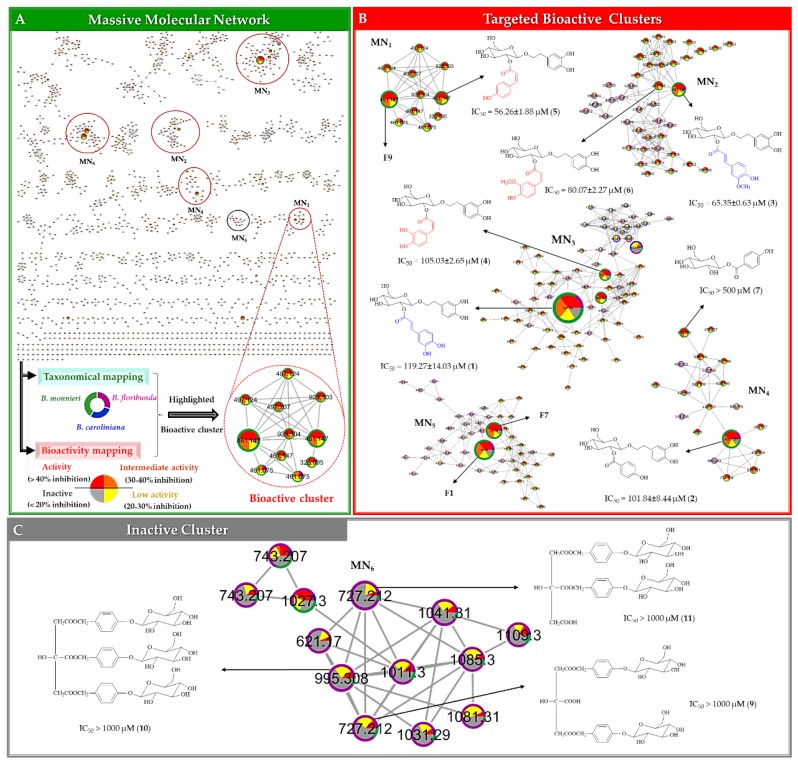
Multi-informational molecular map obtained from the analyses of 59 extracts of three *Bacopa* species mapping with taxonomy and anti-lipid peroxidation activity. Taxonomies of the samples are shown in different colors on the border of each node. Green, blue and purple represent BM, BC and BF, respectively. Bioactivities of the samples are shown in different colors inside each node. The active extracts with an inhibition higher than 40% are represented in red and inactive extracts with less than 40% inhibition are represented in gray. The size of each node is based on the peak height intensity. (**A**) A multi-informative massive molecular network created from MS^2^ datasets. (**B**) Five selected bioactive clusters, MN_1_-MN_5_ with two potential candidates in each cluster, (**C**) an inactive cluster, MN_6_. Chemical structures of the active compounds in Figure 4B,C are expressed with IC_50_ values of anti-lipid peroxidation activity.

Multi-informative MN is a strategy that has previously been demonstrated to effectively prioritize bioactive compounds in natural extract collections [14]. For this, the MS^1^–MS^2^ peaklist was analyzed using such approach in order to visually highlight the clusters of compounds possibly responsible for the observed anti-lipid peroxidation activity. Here, the 4191 features presenting MS^2^ were organized using the GNPS platform to generate a single MN. In this MN, the nodes representing each feature were grouped into 602 clusters by similarity of fragmentation patterns. A multi-informational molecular map was created by merging this MN with biological results and taxonomical information (Figure 4A). All nodes in the MN were color-labeled according to the corresponding lipid peroxidation inhibition level of the extracts (bioactive mapping). This allowed a rapid highlighting of potential bioactive molecular families. Additionally, a taxonomical mapping was applied. The species were differentiated by colored tags on the border of each node (Figure 4A). This additional layout was used to indicate the distribution of plant species for each node. If a given node was most abundantly found in bioactive species, it could be hypothesized that this node was potentially related to an NP responsible for the observed bioactivity of the extract of the corresponding species. Using such mapping, twenty putative bioactive clusters with a minimum of five nodes, corresponding to more than a hundred features were selected by visual inspection based on their dominant red color tags indicating presence in bioactive extracts (Figure S1). The colors of the border (taxonomical origin mapping) suggested that the active nodes were mainly found in *B. monnieri*, while only a few were related to other species. The size of the nodes was based on the MS intensity, which was obtained from an average of the corresponding signal across all samples. In a MN, molecular families tend to cluster together, thus leading to similar ionization behaviours within clusters. Consequently, we hypothesized that the MS intensity of these molecules within a cluster was indicative of their relative abundance. According to this logic, five bioactive clusters (MN_1_–MN_5_, Figure 4B), were further prioritized based on the five largest nodes, leading to a selection of 25 potential bioactive features (Table 1). Among these, seventeen features corresponded to unique molecules, whereas the other neighboring nodes connected to these features were dimeric or adduct forms. Thus, seventeen compounds from MN_1_‒MN_5_ were considered as bioactive candidates from MN (Table 1). An example of unprioritized cluster (MN_6_), potentially linked to non-active metabolites, with dominant grey color tags is also shown in Figure 4C for comparison purposes.

#### 2.2.4. Merging MVA and MN for Bioactive Candidate Prioritization

The S-plot of MVA brings statistical correlation between features and bioactivity but can however be biased by scaling and normalization processes. On the other side, the bioactivity-informed MN approach allows to highlight structural relations between putative bioactive compounds and thus, despite the lack of statistics, allows to indirectly discriminate possible MS artefacts from specialized metabolites features. In order to prioritize unique bioactive molecules from the merging of MVA and MN, common features found in both approaches were highlighted with color tags (see in Table 1) and prioritized as potential bioactive candidates. The two largest nodes found in each five selected clusters (MN_1_‒MN_5_, Figure 4B) represented features also found in the list of putative bioactive candidates in MVA. Therefore, these ten features (**F1**, **F3**, **F6**, **F7, F9**, **F10**, **F12**, **F14**, **F15** and **F17**) were prioritized as potential bioactive compounds for this study (Table 1).

### 2.3. DNP-ISDB Dereplication and Purification of Bioactive Candidates

In the MN, the acquired MS^2^ spectra of each node from the whole MN were matched automatically against GNPS spectral libraries and then annotated against an in silico spectral database build from the Dictionary of Natural Products (DNP-ISDB) as previously described [1] thus providing an identification of level 2 [52]. These spectra were subsequently matched against with a subset of the DNP-ISDB restricted to Plantaginaceae specialized metabolites in order to refine the dereplication results. The top five candidate structures with highest spectral similarity scores were retrieved and the chemical structures for each node was directly visualized within the network using Cytoscape and the ChemViz plugin. The candidate structures for each node were ranked according to their spectral similarity score and the structure with the highest score was reported (Table 1).

Compounds **F1** and **F7** (*m/z* 191.0197 [M − H]¯) were isomers, which were both annotated as idaric acid-1,4-lactone. The other three pairs of isomers i.e., **F3** and **F12** (*m/z* 477.1421 [M − H]¯), **F9** and **F14** (*m/z* 461.1473 [M − H]¯) and **F10** and **F15** (*m/z* 491.1581 [M − H]¯), were proposed to be plantainoside B, 8-*O*-(6’-*O*-trans-coumaroyl-*β*-d-glucopyranosyl)-3,4-dihydroxyphenylethanol and monnieraside II, respectively. The **F6** was predicted to be monnieraside III. The unprioritized features, **F34**–**36**, did not match with MS^2^ spectra libraries from DNP–ISDB, however **F35** was matched against the GNPS spectral library entry parishin A. From these dereplication results, the four pairs of isomers could not be differentiated and two inactive features were given no annotation. Therefore, they may have been new compounds. To establish their structures and evaluate their bioactivity potential, targeted purification of these potential bioactive and inactive compounds was carried out. In order to isolate these compounds, the active extract of *B. monnieri* was fractionated by medium pressure liquid chromatography coupled to an ultraviolet detector (MPLC-UV). The conditions of this separation were first developed by HPLC–UV using a column with identic stationary phase. After this, the analytical HPLC conditions were geometrically transferred to semi–preparative MPLC-UV [53]. All of the MPLC fractions obtained were systematically analyzed by LC-MS. Using the retention time and molecular weight, it was possible to localize the ten potential bioactive candidates (**F1**, **F3**, **F6**, **F7, F9**, **F10**, **F12**, **F14**, **F15** and **F17**) and three unprioritized features (**F34**–**36**). MPLC fractions were further purified by semi–preparative HPLC. As for the separation using MPLC, the conditions of the semi–preparative HPLC were first developed in an analytical method using a column with a similar stationary phase chemistry. After this step, the condition was successfully transferred to the semi–preparative HPLC [54] (Figure S2). In order to avoid loss of resolution, the sample was introduced into the semi–preparative HPLC column by dry load according to a recently developed protocol [55]. Thanks to this approach, it was possible to obtain a high–resolution separation of the majority of the polar compounds, allowing them to be obtained in a high degree of purity. Using this system, seven bioactive candidates prioritized by MVA and MN (compounds **1**–**7**, corresponding to features **F3, F6, F10, F12, F14, F15** and **F17**, respectively) and one compound highlighted MVA only (compound **8**, corresponding to feature **F5**) were isolated. In addition, compounds **9**–**11** (features **F34**–**36**), which were all found in a non–prioritized cluster (MN_6_, Figure 4C) and in an area of the S-plot indicative of potential inactive compounds (lower left corner, Figure 3E), were also isolated.

### 2.4. Identification and Structure Elucidation of Compounds ***1***–***11***

**Figure 5:**
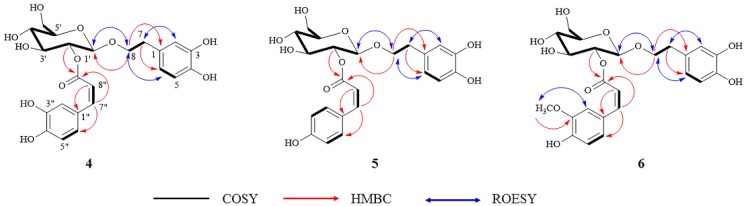
The COSY, HMBC and ROESY correlations of new compounds **4**–**6**.

After purification, all isolated compounds were fully characterized by extensive 2D NMR experiments, which complemented the HRMS^2^ results. Compounds **1**–**3** and **7**–**11**, were identified as plantainoside B (**1**), monnieraside III (**2**), monnieraside II (**3**) [48], 4-hydroxybenzoyl glucose (**7**) [56], 3,4-dihydroxyphenethyl glucoside (**8**) [57], parishin C (**9**), parishin A (**10**) and parishin B (**11**) [58], respectively, by comparing their spectral data with literature. Compounds **4**–**6** were isolated for the first time and identified as new phenylethanoid glycosides: monnieraside IV (**4**), monnieraside V (**5**), and monnieraside VI (**6**). The ^1^H and ^13^C-NMR of **4**–**6** are provided in Table 2 and their COSY, HMBC and ROESY correlations are shown in Figure 5. 

The 2D NMR spectra and HRMS spectra are provided as supplementary data (Figures S3–S21). The structures of seven isolated bioactive candidates are displayed on the prioritized clusters in Figure 4B and structure of the three unprioritized compounds are provided in Figure 4C.

Compound **4** was obtained as a white amorphous powder. The HRESIMS of this compound exhibited a deprotonated molecular ion at *m/z* 477.1420 [M − H]¯ corresponding to the molecular formula C_23_H_26_O_11_ (calcd. 477.1402), indicating an isomer of plantainoside B (**1**). The NMR data of **4** showed close similarities to those of plantainoside B except that the value of the coupling constant between the two ethylenic protons at *δ*_H_ 5.73 (H‒8") and 6.81 (H‒7") of 12.8 Hz indicated a *cis*-form for the caffeoyl group in **4**. The structure of **4** was therefore established as 8-*O*-(2’-*O*-*cis*-caffeoyl-*β*-d-glucopyranosyl)-3,4-dihydroxyphenylethanol (monnieraside IV).

Compound **5** was obtained as a white amorphous powder and it showed a deprotonated molecular ion at *m/z* 461.1473 [M − H]^−^, which was consistent with the molecular formula C_23_H_26_O_10_ (calcd. 461.1453). The NMR data of **5** exhibited a *para*-disubstituted moiety at *δ*_H_ 6.74 (2H, d, *J =* 8.7 Hz, H*‒*3'', H*‒*5''*)* and 7.60 (2H, d, *J =* 8.7 Hz, H*‒*2'', H*‒*6''*)* instead of the tri*-*substituted group of the *cis-*caffeoyl of **4**. The 16 Da mass difference between these two compounds was consistent with this NMR observation. The structure of **5** was established as 8-*O*-(2’-*O*-*cis*-coumaroyl-*β*-d-gluco-pyranosyl)-3,4-dihydroxyphenylethanol (monnieraside V).

The molecular formula of compound **6** (white amorphous powder) was calculated as C_24_H_28_O_11_ by analysis of its HRESIMS (*m/z* 491.1580 [M − H]¯, calcd. 491.1559). The NMR data of **6** showed similarities with those of compound **3** (monnieraside II) both of them being isomers. A *cis*-feruoyl group was present in **6**, as confirmed by the coupling constant value of 12.8 Hz between H‒7" and H‒8" protons**.** The structure of **6** was thus established as 8-*O*-(2’-*O*-*cis*-feruloyl-*β*-d-glucopyranosyl)-3,4-dihydroxyphenylethanol (monnieraside VI).

According to DNP-ISDB dereplication results for the eleven isolated compounds (**1**‒**11**), four compounds; plantainoside B (**1**), monnieraside III (**2**), monnieraside II (**3**) and 4-hydroxybenzoyl glucose (**7**) were correctly annotated as confirmed by NMR results (Table 1). Compound **8** was attributed an incorrect structure (2-(3,5-dihydroxyphenyl)ethanol-3'-*O*-*β*-d-glucopyranoside) by MS^2^ dereplication, however this annotation was related to the structure later established by NMR (3,4-dihydroxyphenethyl glucoside). In addition, DNP-ISDB dereplication proposed the structure of **F9** and **5** (*m/z* 461.1473 [M − H]¯) as 8-*O*-(6’-*O*-*trans*-coumaroyl-*β*-d-glucopyranosyl)-3,4-dihydroxy-phenylethanol. However, NMR data of **5** suggested it as 8-*O*-(2’-*O*-*cis*-coumaroyl-*β*-d-glucopyranosyl)-3,4-dihydroxyphenylethanol. This indicated that the structure of **F9** could indeed be the dereplicated *trans–*isomer. Furthermore, previous observation showed that the *trans*-isomers of phenylpropanoid derivatives (compounds **1** and **3**) had shorter retention time than their *cis*-isomers counterpart (compounds **4** and **6**). The same phenomenon was also observed for **F9** and **5**. Consequently, **F9** could therefore correspond to as 8**-***O*-(2’-*O*-*trans*-coumaroyl-*β*-d-glucopyranosyl)-3,4-dihydroxy-phenylethanol. In addition, we found that the annotation against GNPS spectral libraries of compound **10** (parishin A) was correct, as confirmed by NMR structural elucidation. In MN_6_, the annotation of node *m/z* 995.3078 [M − H]¯ with parishin A and the mass difference of 268.0950 with two neighboring nodes at *m/z* 727.2121 [M − H]¯, RT 1.19 min and 727.2124 [M − H]¯, RT 1.10 min indicated a possible loss of the 4-(*β*-d-glucopyranosyloxy)benzyl alcohol moiety (–C_13_H_16_O_6_, calcd. 268.0946) present on parishin A. After isolation and NMR identification structural elucidation, **9** and **11** were indeed found to be parishin C and parishin B, respectively, illustrating the interest of MN for dereplication purposes.

### 2.5. Evaluation of the Anti-Lipid Peroxidation Activity of the Isolated Compounds

In order to verify the bioactivity potential of the compound prioritized by the combination of MVA and multi-informative MN, the seven isolated compounds were tested for their anti-lipid peroxidation activity with the TBAR assay. Six compounds, **1**–**6**, showed inhibitory activity with IC_50_ values < 120 µM and one compound, **7**, had lower activity (IC_50_ > 500 µM) (Table 1). The positive control (Trolox) showed an IC_50_ value of 13.92 ± 0.32 µM. For this study, we defined compounds presenting IC_50_ values not higher than 10-fold of the control’s IC_50_ value as active compounds. 

Some prioritized features (**F1**, **F7** and **F9**) could not be isolated, their bioactivity potential is however discussed according to the following evidences. Features **F1** and **F7** were proposed to be idaric acid-1,4-lactone and its isomer by MS^2^. The d enantiomer, d-glucaric-1,4-lactone, has been reported to exert anti-lipid peroxidation and anti-oxidant activities [59]. Compound **F9** was also likely to exhibit anti-lipid peroxidation activity similar to its isomer (**5**), in the same fashion as isomeric compounds **1**/**4** and **3**/**6** also shared the same range of anti-lipid peroxidation activity. Given the structure similarity of **1**–**6**, their inhibitory activities were compared. The activity of the compounds tends to decrease when C3'' was substituted with methoxy (**3**, **6**) and hydroxy groups (**1**, **4**), respectively (Figure 4B). Such substitutions might reduce the ability of the compounds to protect the oxidation of Fe^2+^ to Fe^3+^ in the lipid peroxidation process.

To verify that the proposed merging of MVA and MN decreased the numbers of false positive candidate compounds, two features **F5** and **F16** (identified to bacopaside I using standard comparison) highlighted by MVA only were assayed. Both showed low levels of activity with IC_50_ values > 500 µM and > 1000 µM, respectively. This indicated that the combination of both prioritization approaches could help to further refine the prioritization process and lower the rate of false positives isolation.

Additionally, three unprioritized compounds **9**–**11** were isolated and their anti-lipid peroxidation activity assayed. We found that these three compounds displayed very low inhibition of lipid peroxidation with the IC_50_ values > 1000 µM, indicating that the employed strategy was effective to highlight bioactive compounds from complex mixtures of NPs prior to isolation.

**Figure 6:**
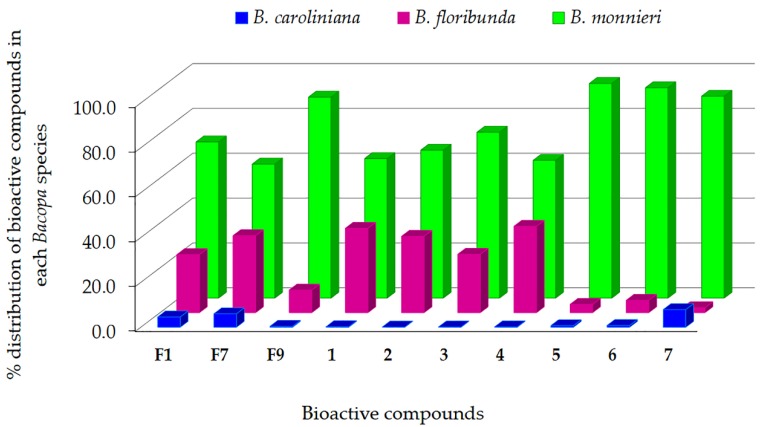
Percentage distribution of ten bioactive compounds in each *Bacopa* species, calculated by division of the average MS signal intensity of the compound in each species by a sum of signal intensities of the compound in three *Bacopa* species ×100.

Further investigations of ten prioritized bioactive features **F1**, **F7**, **F9** and compounds **1**–**7** revealed that they were differently distributed (% of MS intensities) in the three *Bacopa* species (Figure 6). The highest mean distribution of these compounds was observed in *B. monnieri* (77%), followed by *B. floribunda* (21%). This result agreed with the finding that *B. monnieri* had higher anti-lipid peroxidation activity than *B. floribunda* (Figure 2), suggesting that these compounds are the bioactives responsible for the anti–lipid peroxidation effects observed in *B. monnieri* and *B. floribunda*. Even though *B. caroliniana* presented the lowest level of these active compounds (~2%), it still showed some inhibition of lipid peroxidation, which could indicate the presence of other bioactive compounds in the plant.

The biological evaluation of the compounds prioritized by merging MVA and MN, indicated the validity of the approach. However, some bioactive compounds could be missed since the selection in MN was partly based on MS signal intensity, which is non–quantitative and highly dependent on the chemical structure of the analytes. The hyphenation of MS analytical platforms with universal detectors such as evaporative light scattering detector (ELSD) should offer a more accurate view of the precise quantitative repartition of metabolites within complex matrices, hence enhancing the power of MS–based prioritization approaches.

## 3. Materials and Methods

### 3.1. Chemicals and Plant Materials

All chemicals used were of analytical grade and obtained from Sigma-Aldrich (St. Louis, MO, USA). All solvents were HPLC and LC-MS grades. Acetonitrile (ACN), methanol (MeOH) and formic acid were purchased from Merck (Darmstadt, Germany). Water was purified by a Milli–Q purification system from Millipore (Bedford, MA, USA).

Three *Bacopa* species i.e., 36 samples of *B. monnieri* (BM1‒12), 12 samples of *B. caroliniana* (BC1‒4), and 11 samples of *B. floribunda* (BF1 from nature and BF2‒11 from tissue culture) were collected from different regions and seasons. Only collected BM and BC samples were planted under the same growing conditions in the Faculty of Pharmaceutical Sciences, Naresuan University and subsequently harvested during different seasons in 2017: January (represented winter), April (summer), and July (rainy season) to enable an evaluation of the effect of these seasonal conditions. These plants species were identified by Dr. Pranee Nangngam and their voucher specimens (Saesong001‒17) have been deposited at Department of Biology, Faculty of Science, Naresuan University. The codes and information regarding the samples are presented in Table 3.

The shoot part (10 cm) of each *Bacopa* sample was collected based on a previous method [45]. Then it was cleaned and dried at 50 °C in a hot air oven for 24 h. The dried plants were crushed and passed through a 60 mesh sieve and stored in plastic containers under refrigeration at −20 °C until used.

### 3.2. Sample Preparation

Metabolites of *Bacopa* were extracted by adding 1 mL of 70%MeOH to a powdered sample (20 mg). The solution was then sonicated at room temperature for 15 min and filtered through a 0.45 µm nylon filter. Each extract solution was analyzed by UPHLC-HRMS^2^ and tested for anti-lipid peroxidation activity in parallel.

### 3.3. TBAR Assay

Lipid peroxidation inhibitory activity was tested by TBARs assay, with minor modification to a previous study [50]. In this process, 20 µL of sample and 140 µL of homogenate rat brain (contained 5.72 mg/mL total protein) were mixed and incubated at 37 °C for 30 min. Then, 20 µL of 4 mM Fe_2_SO_4_ and 2 mM ascorbic acid were added to the mixture solution and incubated at 37 °C for 1 h. After incubation, 200 µL of TBARs reagent (40% trichloroacetic acid, 1.4% thiobarbituric acid, and 8% HCl) was added and incubated at 90 °C for 60 min. The mixture was then allowed to cool to room temperature and centrifuged at 10,000 rpm at 4 °C for 5 min to pelletize the precipitated protein. The absorbance of the supernatant was read at 530 nm by a microplate reader (BioTek Instruments, Winooski, Vermont, USA). The inhibition was calculated by comparison with the negative control. The homogenized rat brain in this assay was prepared in 1x PBS buffer (pH 7.4). The protein content in the homogenized rat brain was measured using a bicinchoninic acid (BCA) assay [60].

### 3.4. UHPLC-ESI-HRMS^2^ Analysis

The UHPLC−HRMS^2^ was carried out on a Waters Acquity UPLC IClass system system interfaced to a Q-Exactive Focus mass spectrometer (Thermo Scientific, Bremen, Germany), using a heated electrospray ionization (HESI-II) source. Chromatographic separation was performed on a Waters BEH C18 column (50 × 2.1 mm, 1.7 μm), the mobile phase consisted of 0.1% formic acid in water (A) and 0.1% formic acid in acetonitrile (B), the flow rate was 600 μL/min, the injection volume was 1 μL, and the linear gradient elution initially increased from 5–100% B for 7 min, followed by isocratic conditions at 100% B for 1 min, and then decreased to 5% B for the final step for 2 min. The negative ionization mode was applied in this study because the molecular ion peak of the most important metabolites could not be observed in positive ion mode. The optimized HESI-II parameters were set as follows: source voltage, 3.5 kV; sheath gas flow rate (N_2_), 48 units; auxiliary gas flow rate, 11 units; spare gas flow rate, 2.0 units; capillary temperature, 300 °C, S-Lens RF Level, 55. The mass analyzer was calibrated using a mixture of caffeine, methionine-arginine-phenylalanine-alanine-acetate (MRFA), sodium dodecyl sulfate, sodium taurocholate, and Ultramark 1621 in an acetonitrile/methanol/water solution containing 1% formic acid by direct injection. The data-dependent MS/MS events were performed on the three most intense ions detected in full scan MS (Top3 experiment). The MS/MS isolation window width was 2 Da, and the normalized collision energy (NCE) was set to 35 units. In data-dependent MS/MS experiments, full scans were acquired at a resolution of 35,000 fwhm (at *m/z* 200) and MS/MS scans at 17,500 fwhm, both with a maximum injection time of 50 ms. After being acquired in a MS/MS scan, parent ions were placed in a dynamic exclusion list for 3.0 s. All samples were performed by UHPLC-HRMS^2^ in one batch and a single pool of all samples was used as a quality control (QC). The QC sample was processed to monitor the reproducibility and stability of the system, which was injected at the beginning, then once every ten tested samples, and at the end of the batch analysis.

### 3.5. MZmine data preprocessing

The UHPLC−HRMS^2^ raw data were converted to .mzXML format using MSConvert software, part of the ProteoWizard package and processed with MZmine version 2.32. Six main steps, consisting in mass detection, chromatogram building, deconvolution, isotopic peak grouping, alignment and gap filling, were carried. The mass detection was set in centroid mode and the noise level was kept at 1 × 10^6^ for MS^1^ and 0 for MS^2^. The ADAP chromatogram builder was selected and run using a minimum group size in number of scans of 5, minimum height of 1 × 10^6^, and *m/z* tolerance of 0.001 Da (or 5 ppm). The chromatogram deconvolution was set as follows: wavelets (ADAP) was used as the algorithm for peak recognition, *m/z* and RT range for MS^2^ scan pairing were 0.3 Da and 0.1 min, S/N threshold was 20, minimum feature height was 1 × 10^6^, coefficient/area threshold was 110, peak duration range was 0.01–1.0 min, and the RT wavelets range was 0.001–0.04. Chromatograms were then deisotoped by isotopic peaks a grouper algorithm with a *m/z* tolerance of 0.001 Da and an RT tolerance of 0.05 min. Peak alignment was carried out using a join aligner, with *m/z* tolerance set at 0.001 Da, absolute RT tolerance at 0.05 min, and weight for *m/z* and RT at 30. The missing peaklist after alignment was filled by gap filling of same RT and *m/z* range gap filler module with a *m/z* tolerance of 0.001 Da. After gap filling, all peaklists were done with identification of adduct search, complex search, and molecular formula prediction. This resulted in a peaklist of 6082 features which was further filtered to a peaklist of 4191 features having an associated data dependent MS^2^ spectra. This resulting peaklist of 4191 features was exported as input for the MVA (MS^1^ data only) and for MN generation (MS^1^ and MS^2^ data).

### 3.6. Multivariate Analysis

After data treatment with MZmine, a three-dimensional data matrix comprising of retention time, *m/z* value and peak height were analyzed by SIMCA-P software (version 13.0, Umerics, Umea, Sweden). Pareto-scaling was applied to normalize data for PCA and OPLS analysis. In addition, R^2^ and Q^2^ (cum) were used for model evaluation. Values of both parameters close to 1.0 indicated a good fitness for the created model. OPLS, a supervised multivariate statistical method, was completed with percent inhibition of lipid peroxidation activity as the Y input. The features with potential bioactivity from S-plot in OPLS model were selected based on their p[1] values.

### 3.7. Molecular Networking Analyses

The MN of MS^2^ spectra of the *Bacopa* species was generated using the online workflow of the Global Natural Products Social Molecular Networking (GNPS). The MS^2^ spectra were then clustered with MS-Cluster with a parent mass tolerance at 0.02 Da and a fragment ion mass tolerance at 0.02 Da to create consensus spectra, and consensus spectra containing less than two spectra were discarded. A network was then created, where edges were filtered to have a cosine score above 0.7 and more than 6 matching peaks. Furthermore, the edges between two nodes were kept in the network if each of the nodes appeared in each other’s respective top 10 most similar nodes. The spectra in the network were automatically searched against GNPS spectral libraries and then against DNP-ISDB according to a previously described methodology [1]. ChemViz 1.3 plugin (freely available at [61]) was used to display the structure of the dereplication hits directly within Cytoscape 3.6.1. The generated MN in this study can be seen in [62] and the MASSIVE datasets contained all raw data was provided in the link of [63].

### 3.8. Purification of Candidate Bioactive Compounds

#### 3.8.1. Extraction Procedure

The dried powder of BM4 (100 g) was macerated three times with MeOH and shaken for 24 h to give 24.2 g of MeOH extract. The polar substances (sugar) of the extract were removed using solid phase extraction prior to purification using the following protocol. The 200 g of C18 (ZEOprep^®^ 60 C18, 40–63 µm) was packed in a column and activated by MeOH (1 L), followed by conditioning with water (1 L). Then, 2 g of Brahmi extract in 10 mL water was loaded and the column was washed with water (1 L) to remove polar substances. Remaining compounds were finally eluted with MeOH (1 L).

#### 3.8.2. Purification Methods

The isolation steps of candidate compounds were performed by MPLC and followed by semi–preparative HPLC. A system of MPLC was carried out on an LC instrument conducted with a 681-pump module C‒615, UV-Vis module C‒640, and a fraction collector module C‒660 (Buchi, Flawil, Switzerland). Fractionation was performed with an ZEOprep^®^ C18 column (70 × 460 mm, 15–25 μm) with elution of 0.1% formic acid in water (A) and 0.1% formic acid in acetonitrile (B). The gradient elution started from 0‒20 min of 35% B and then increased to 100% B for 284 min. The flow rate was 20 mL/min. This condition was first optimized on an analytical HPLC column (250 × 4.6 mm i.d., 15–25 μm, Zeochem, Uetikon am See, Switzerland) packed with the same stationary phase and then geometrically transferred to the preparative scale [53]. The extract was introduced into the MPLC column by dry injection by mixing 5.62 g of the extract with 18.30 g of the Zeoprep C18 stationary phase (40–63 μm, Zeochem). The mixture was conditioned in a dry-load cell (11.5 × 2.7 cm i.d.) and it was connected subsequently between the pumps and the MPLC column. Twenty-five fractions were collected by peak-based detection under UV at 205, 254 and 366 nm. When there were no peaks, 250 mL of each of the fractions was collected. The candidate compounds in the fractions were tracked down by LC-MS using the same conditions as mentioned in session 3.4.

The candidate bioactive compounds (**1**–**8**) and inactive compounds (**9**–**11**) were isolated from fraction 3 of MPLC using semi−preparative HPLC, which was performed on a HPLC-UV instrument with SPD-20A UV-Vis, a LC-20AP Pump, a FRC-10A fraction collector and a sample injector (Shimadzu, USA). The separation was carried out on an XBridge C18 OBD prep column (19 × 250 mm, 5 μm, Waters, Milford, MA, USA) with a guard column (4 × 20 mm, 5 μm), using an isocratic system of 0.1% formic acid in water and in acetonitrile at ratios of 86 and 14 as mobile phase. The separation time was 65 min with a post run of 10 min and the flow rate set at 17 mL/min. This semi−preparative HPLC condition was optimized on an analytical HPLC using a column with a similar stationary phase (XBridge C18, 4.6 × 250 mm, 5 μm) and then the optimum condition was geometrically transferred to the semi-preparative scale [54]. In order to avoid loss of resolution, the sample was loaded into the column by dry loading according to a recently developed protocol [55], which made it possible to obtain a high−resolution separation of the majority of the polar compounds to ensure a high degree of purity. Using this preparative system, eighty-four fractions were collected by peak-based detection under UV absorption of 205, 254 and scan 200–600 nm and volume based collection (8 mL of each fraction). All collected fractions were dried by speed vacuum (Genevac HT-4X, Genevac Ltd., North Carolina, USA). Isolation was achieved and afforded candidate bioactive compounds of **1** (8.5 mg), **2** (2.2 mg), **3** (0.9 mg), **4** (1.4 mg) **5** (0.1 mg) **6** (0.6 mg) and **7** (0.8 mg) and **8** (3.0 mg) and three inactive compounds of **9** (0.4 mg), **10** (3.0 mg) and **11** (1.2 mg). The purity and structure elucidation of each isolated compound was checked with HPLC, MS and NMR.

#### 3.8.3. Identification Procedures

The NMR spectra of each isolated compound was recorded on a Bruker Avance Neo 600 MHz spectrometer equipped with a QCI 5mmCryoprobe and a SampleJet automated sample changer (Bruker BioSpin, Rheinstetten, Germany) (600). Chemical shifts (*δ*) were recorded in parts per million in methanol-*d*_4_ using the residual solvent signal (*δ*_H_ 3.31; *δ*_C_ 49.0) as internal standards for ^1^H and ^13^C-NMR, respectively. Mass spectrometric data were obtained on a Waters Acquity UPLC IClass system system interfaced to a Q-Exactive Focus mass spectrometer (Thermo Scientific).

## 4. Conclusions

In this work, the integration of MVA and multi-informative MN based on LC–HRMS^2^ metabolite profiling with bioactivity data was proven to be an efficient way to identify bioactive constituents in closely related plant extracts. The data generated allowed a rapid prioritization of bioactive compounds on a specific target from crude *Bacopa* extracts. Thanks to this approach the potential bioactivity for individual compounds could be anticipated prior to any physical separation process. This allowed the targeted isolation of six phenylethanoid glycosides **1**–**6** of *Bacopa* species with lipid-peroxidation inhibitory activity three of them being novel compounds i.e., monnieraside IV (**4**), monnieraside V (**5**) and monnieraside VI (**6**).

Additionally, the results in MVA and MN showed significant difference between Brahmi samples harvested in summer and other seasons in term of overall biological activity and amount of bioactive compounds. To our knowledge, Brahmi is collected throughout the year in Thailand and, based on our study, seasonal effects are important to consider and might affect the medicinal properties claimed for Brahmi. The described bioactive compounds could be used as biomarkers for quality control of this plant.

## Figures and Tables

**Table 1:** The nineteen bioactive candidate features (**F1**–**F19**) ranked by p[1] value from S-plot of MVA and twenty-five bioactive candidates selected from prioritized clusters of MN (MN_1_–MN_5_). Ten potential bioactive compounds prioritized from the merging of MVA and MN approach are highlighted (one color per cluster). IC_50_ values of the compounds on anti-lipid peroxidation activity are expressed in µM and as an average from triplicate experiments ± standard deviation.

ID	*m/z*	RT (min)	p[1] Value*	Selected Bioactive Cluster	Chemical Formula	Δ ppm	Dereplicated Compounds (MSMS Based Identification)**	Isolated Compounds (NMR Identification)	Anti-Lipid Peroxidation Activity (IC_50_ (µM))
**F1**	191.0197[M − H]¯	0.25	0.239	MN_5_	C_6_H_8_O_7_	0.14	Idaric acid-1,4-lactone		
**F2**	217.0487[M + Cl]¯	0.23	0.207	NS	C_6_H_14_O_6_	−1.20	Adduct of **F18**		
**F3**	477.1420[M − H]¯	1.38	0.204	MN_3_	C_23_H_26_O_11_	−3.91	Plantainoside B^b^	Plantainoside B (**1**)	119.27 ± 14.03
**F4**	377.0873[M − H]¯	0.24	0.183	NS	C_18_H_18_O_9_	1.08	4,8-Dihydroxy-1,2,3,6,7-pentamethoxy-9H-xanthen-9-one		
**F5**	315.1099[M − H]¯	0.60	0.166	NS	C_14_H_20_O_8_	−4.31	2-(3,5-Dihydroxyphenyl)ethanol-3'-*O*-*β*-d-glucopyranoside	3,4-dihydroxyphenethyl glucoside (**8**)	>500
**F6**	435.1313[M − H]¯	1.28	0.165	MN_4_	C_21_H_24_O_10_	−3.74	Monnieraside III^a^	Monnieraside III (**2**)	101.84 ± 8.44
**F7**	191.0197[M − H]¯	0.31	0.163	MN_5_	C_6_H_8_O_7_	0.14	Idaric acid-1,4-lactone isomer		
**F8**	955.2917[2M − H]^−^	1.38	0.156	MN_3_	C_23_H_26_O_11_	−4.91	Dimer of **F3**		
**F9**	461.1473[M − H]¯	1.60	0.154	MN_1_	C_23_H_26_O_10_	−4.29	8-*O*-(6’-*O*-*trans*-Coumaroyl-*β*-d-glucopyranosyl)-3,4-dihydroxyphenylethanol		
**F10**	491.1580[M − H]¯	1.67	0.152	MN_2_	C_24_H_28_O_11_	−4.51	Monnieraside II^a^	Monnieraside II (**3**)	65.35 ± 0.63
**F11**	1063.4467[M − H]¯	3.07	0.128	NS	C_49_H_76_O_23_S	−3.92	Unidentified^c^		
**F12**	477.1420[M − H]¯	1.53	0.119	MN_3_	C_23_H_26_O_11_	−3.91	Plantainoside B^b^	Monnieraside IV^e^ (**4**)	105.03 ± 2.65
**F13**	219.0458[M − H]¯	0.23	0.118	NS	C_15_H_8_O_2_	−3.41	Unidentified^c^		
**F14**	461.1473[M − H]¯	1.71	0.115	MN_1_	C_23_H_26_O_10_	−4.29	8-*O*-(6’-*O*-*trans*-Coumaroyl-*β*-d-glucopyranosyl)-3,4-dihydroxyphenylethanol	Monnieraside V^e^ (**5**)	56.26 ± 1.88
**F15**	491.1579[M − H]¯	1.78	0.113	MN_2_	C_24_H_28_O_11_	−4.31	Monnieraside II^a^	Monnieraside VI^e^ (**6**)	80.07 ± 2.27
**F16**	977.4458[M − H]¯	3.00	0.112	NS	C_46_H_74_O_20_S	−3.75	Bacopaside I^a^		>1000^f^
**F17**	299.0786[M − H]¯	0.78	0.110	MN_4_	C_13_H_16_O_8_	−4.54	4-Hydroxybenzoyl glucose^b^	4-hydroxybenzoyl glucose (**7**)	>500
**F18**	181.0717[M − H]¯	0.23	0.107	NS	C_6_H_14_O_6_	−0.21	Mannitol^d^		
**F19**	631.2274[M − H]¯	0.60	0.104	NS	C_46_H_32_O_3_	0.74	Unidentified^c^		
**F20**	497.1243[M + Cl]¯	1.58	0.033	MN_1_	C_23_H_26_O_10_	−4.63	Adduct of **F9**		
**F21**	461.0749[M − H]¯	1.56	0.003	MN_1_	C_21_H_18_O_12_	−5.09	3',4',5,7-Tetrahydroxyflavone5-*O*-*β-*d-glucurono-pyranoside		
**F22**	497.1243[M + Cl]¯	1.71	0.029	MN_1_	C_23_H_26_O_10_	−4.63	Adduct of **F14**		
**F23**	521.1685[M − H]¯	1.66	0.053	MN_2_	C_25_H_30_O_12_	−3.93	Aucubigenin-10-*O*-(4-hydroxy-3-methoxy-cinnamoyl), 1-*O*-*β*-D-glucopyranoside^b^		
**F24**	983.3235[2M − H]¯	1.67	0.084	MN_2_	C_24_H_28_O_11_	−4.53	Dimer of **F10**		
**F25**	527.1347[M + Cl]¯	1.67	0.030	MN_2_	C_24_H_28_O_11_	−4.05	Adduct of **F10**		
**F26**	341.0893[M − H]¯	0.66	0.064	MN_3_	C_15_H_18_O_9_	−4.37	Chaenorrhinoside^b^		
**F27**	477.1421[M − H]¯	1.62	0.043	MN_3_	C_23_H_26_O_11_	−3.90	Plantainoside B^b^		
**F28**	871.2706[2M − H]¯	1.29	0.077	MN_4_	C_21_H_24_O_10_	−4.57	Dimer of **F6**		
**F29**	471.1084[M + Cl]¯	1.29	0.033	MN_4_	C_21_H_24_O_10_	−4.36	Adduct of **F6**		
**F30**	599.1642[2M − H]¯	0.79	0.068	MN_4_	C_13_H_16_O_8_	−4.07	Dimer of **F17**		
**F31**	421.0046[M − H]¯	0.31	0.008	MN_5_	C_17_H_10_O_13_	0.63	Unidentified^c^		
**F32**	405.0308[M − H]¯	0.31	0.057	MN_5_	C_14_H_14_O_14_	0.69	Unidentified^c^		
**F33**	191.0198[M − H]¯	0.38	0.027	MN_5_	C_6_H_8_O_7_	−0.38	Idaric acid-1,4-lactone		
**Three isolated inactive compounds selected from inactive cluster (MN_6_) of the MN and in S-plot of MVA **									
**F34**	727.2125[M − H]¯	1.20	−0.026	MN_6_	C_32_H_40_O_19_	−4.67	Unidentified^c^	Parishin C (**9**)	>1000
**F35**	995.3078[M − H]¯	1.40	−0.037	MN_6_	C_45_H_56_O_25_	−4.03	Parishin A^d^	Parishin A (**10**)	>1000
**F36**	727.2123[M − H]¯	1.12	−0.060	MN_6_	C_32_H_40_O_19_	−4.40	Unidentified^c^	Parishin B (**11**)	>1000

* The values obtained from S-plot of OPLS and ** DNP-ISDB in silico fragmented results unless specified (Top 1 result only are reported). ^a,b^ The compound has been previously reported in *B. monnieri* and Plantaginaceae family, respectively, ^c^ No matching with DNP-ISDB or GNPS libraries, ^d^ Annotated compound from GNPS spectral libraries, ^e^ New compound and ^f^ Standard compound. NS: not selected from bioactive clusters (MN_1_–MN_5_) but from other clusters in MN.

**Table 2:** ^1^H- and ^13^C-NMR (600/151 MHz, in CD_3_OD) of **4**–**6**.

Position	Monnieraside IV (4)		Monnieraside V (5)		Monnieraside VI (6)	
	*δ*_C_	*δ*_H_ (*J* in Hz)	*δ*_C_	*δ*_H_ (*J* in Hz)	*δ*_C_	*δ*_H_ (*J* in Hz)
1	131.5		131.3		131.2	
2	117.0	6.61, d (2.1)	116.8	6.62, d (2.1)	116.6	6.61, d (2.1)
3	146.0		145.8		145.8	
4	144.6		144.4		144.3	
5	116.3	6.61, d (8.1)	116.1	6.61, d (8.1)	116.2	6.60, d (8.1)
6	121.4	6.49, dd (8.1, 2.1)	121.1	6.50, dd (8.1, 2.1)	121.1	6.48, dd (8.1, 2.1)
7	36.5	2.68, t (7.2)	36.3	2.68, m	36.3	2.67, t (7.1)
8	71.9	3.64, dt (9.8, 7.2) 4.00, dt (9.8, 7.2)	71.5	3.63, dt (9.4, 7.3) 4.01, dt (9.4, 6.9)	71.6	3.63, dt (9.8, 7.1) 4.00, dt (9.8, 7.1)
1'	102.3	4.45, d (8.1)	102.0	4.44, d (8.1)	102.1	4.46, d (8.0)
2'	74.9	4.79, dd (9.3, 8.1)	74.6	4.79, dd (9.3, 8.1)	74.7	4.79, dd (9.3, 8.0)
3'	76.2	3.51, t (9.3)	75.9	3.50, t (9.3)	76.0	3.52, t (9.3)
4'	71.8	3.38, t (9.3)	71.5	3.38, t (9.3)	71.5	3.38, t (9.3)
5'	78.1	3.29 (overlapped)	77.9	3.28 (overlapped)	77.9	3.30 (overlapped)
6'	62.6	3.69, dd (12.0, 5.7) 3.88, dd (12.0, 2.3)	62.4	3.69, dd (11.9, 5.8) 3.88, dd (11.9, 1.8)	62.4	3.69, dd (12.0, 5.8) 3.88, dd (12.0, 2.3)
1"	128.2		127.6		127.9	
2"	118.7	7.38, d (2.1)	133.4	7.60, d (8.7)	114.8	7.72, d (2.0)
3"	145.7		115.5	6.74, d (8.7)	148.0	
4"	148.3		159.8		149.1	
5"	115.7	6.72, d (8.2)	115.5	6.74, d (8.7)	115.4	6.76, d (8.2)
6"	125.1	7.08, dd (8.2, 2.1)	133.4	7.60, d (8.7)	126.4	7.12, dd (8.2, 2.0)
7"	145.1	6.81, d (12.8)	144.6	6.88 d (12.7)	145.1	6.87, d (12.8)
8"	116.7	5.73, d (12.8)	116.6	5.76, d (12.7)	116.5	5.77, d (12.8)
9"	167.4		167.3		167.1	
OCH_3_					56.2	3.85, s

**Table 3:** The geographical details of the *Bacopa* samples collected in this study. Samples 1–16 were collected in 3 seasons i.e., winter, summer, and rainy season (48 samples). Only sample 17 was collected in summer (1 sample). Plant tissue cultures of *B. floribunda* (samples 18–27) were collected in April, 2017 (10 samples).

No.	Code	*Bacopa* spp.	Sources
1	BM1	*B*. *monnieri*	Perth, Australia
2	BM2	*B*. *monnieri*	Wat Phra Sri Mahathat, Bangkok, Thailand
3	BM3	*B*. *monnieri*	Samphan garden, Nakhon Pathom, Thailnd
4	BM4	*B*. *monnieri*	Naresuan University, Phitsanulok, Thailand
5	BM5	*B*. *monnieri*	Kasetsart University, Bangkok, Thailand
6	BM6	*B*. *monnieri*	Nakhon Nayok, Thailand
7	BM7	*B*. *monnieri*	Chatuchak Market, Bangkok, Thailand
8	BM8	*B*. *monnieri*	Ayutthaya, Thailand
9	BM9	*B*. *monnieri*	Fukuoka, Japan (originated in India)
10	BM10	*B*. *monnieri*	Siriraj hospital, Bangkok, Thailand
11	BM11	*B*. *monnieri*	Chatuchak Market, Bangkok, Thailand
12	BM12	*B*. *monnieri*	Phetchabun, Thailand (originated in India)
13	BC1	*B*. *caroliniana*	Naresuan University, Phitsanulok, Thailand
14	BC2	*B*. *caroliniana*	Nakhon Nayok, Thailand
15	BC3	*B*. *caroliniana*	Chiang Mai, Thailand
16	BC4	*B*. *caroliniana*	Bangkok, Thailand
17	BF1	*B*. *floribunda*	Sakolnakorn, Thailand
18–27	BF2 to BF11	*B*. *floribunda*	Plant tissue cultures obtained from Department of Biology, Faculty of Science, Naresuan University

## Supplementary Materials

The following are available online. Figure S1: Twenty candidate bioactive clusters observed by visual inspection based on dominant red color tag and five selected bioactive clusters in red square box (MN_1_‒MN_5_) were nominated based on node size; Figure S2: Representative HPLC chromatograms from method transfer between HPLC (A) and semi-preparative HPLC (B) for separation of compounds **1**–**11** in fraction 3 (from MPLC) of *B. monnieri* extract; Figure S3: HRESIMS spectrum of compound **4** (negative ionization); Figure S4: ^1^H-NMR spectrum of compound **4** in CD_3_OD at 600 MHz; Figure S5: COSY NMR spectrum of compound **4** in CD_3_OD; Figure S6. ^13^C‒DEPTQ NMR spectrum of compound **4** in CD_3_OD at 151 MHz; Figure S7: Edited‒HSQC-NMR spectrum of compound **4** in CD_3_OD; Figure S8: HMBC-NMR spectrum of compound **4** in CD_3_OD; Figure S9: ROESY NMR spectrum of compound **4** in CD_3_OD; Figure S10: HRESIMS spectrum of compound **5** (negative ionization); Figure S11: 1H-NMR spectrum of compound **5** in CD_3_OD at 600 MHz; Figure S12: COSY NMR spectrum of compound **5** in CD_3_OD; Figure S13: Edited‒HSQC-NMR spectrum of compound **5** in CD_3_OD; Figure S14: HMBC-NMR spectrum of compound **5** in CD_3_OD; Figure S15: ROESY NMR spectrum of compound **5** in CD_3_OD; Figure S16: HRESIMS spectrum of compound **6** (negative ionization); Figure S17: ^1^H-NMR spectrum of compound **6** in CD_3_OD at 600 MHz; Figure S18: COSY NMR spectrum of compound **6** in CD_3_OD; Figure S19: Edited‒HSQC-NMR spectrum of compound **6** in CD_3_OD; Figure S20: HMBC-NMR spectrum of compound **6** in CD_3_OD; Figure S21: ROESY NMR spectrum of compound **6** in CD_3_OD.
