# Supplementary material for: Discovery of Lipid Peroxidation Inhibitors from Bacopa Species Prioritized through Multivariate Data Analysis and Multi-Informative Molecular Networking
Source: Molecules. 2019 Aug 17;24(16):2989. doi: 10.3390/molecules24162989 (PMC6719142; doi:10.3390/molecules24162989)
Supplement: Supplementary file 1 [file molecules-24-02989-s001.pdf]

Discovery of lipid peroxidation inhibitors from *Bacopa* species prioritized through multivariate data analysis and multi–informative molecular networking

Tongchai Saesong [1], Pierre-Marie Allard [2], Emerson Ferreira Queiroz [2], Laurence Marcourt [2], Nitra Nuengchamnong [3], Prapapan Temkitthawon [1], Nantaka Khorana [4], Jean-Luc Wolfender [2,*] and Kornkanok Ingkaninan [1,*]

[1] Department of Pharmaceutical Chemistry and Pharmacognosy, Faculty of Pharmaceutical Sciences and Center of Excellence for Innovation in Chemistry, Naresuan University, Phitsanulok 65000, Thailand; tongchai_saesong@hotmail.com (T.S.); prapapantem@gmail.com (P.T.)

[2] School of Pharmaceutical Sciences, EPGL, University of Geneva, University of Lausanne, CMU Rue Michel Servet 1, 1211 Geneva 4, Switzerland; Pierre-Marie.Allard@unige.ch (P.-M.A.); Emerson.Ferreira@unige.ch (E.-F.Q.); laurence.marcourt@unige.ch. (L.M.)

[3] Science Lab Center, Faculty of Science, Naresuan University, Phitsanulok 65000, Thailand; nitra_nuengchamnong@yahoo.com (N.N.)

[4] Division of Pharmaceutical Sciences, School of Pharmaceutical Sciences, University of Phayao, Phayao 56000, Thailand; nantaka@hotmail.com (N.K.)

* Correspondence: E-mail addresses: k_ingkaninan@yahoo.com (K.I.);jean-luc.wolfender@unige.ch. (J.-L.W.)

Supporting information

Table of Contents

Figure S1. Twenty candidate bioactive clusters observed by visual inspection based on dominant red color tag and five selected bioactive clusters in red square box (MN_1–MN_5) were nominated based on node size. 3

Figure S2. Representative HPLC chromatograms from method transfer between HPLC (A) and semi-preparative HPLC (B) for separation of compounds **1**–**11** in fraction 3 (from MPLC) of *B. monnieri* extract 4

Figure S3. HRESIMS spectrum of compound **4** (negative ionization) 4

Figure S4. ^{1}H NMR spectrum of compound **4** in CD_3OD at 600 MHz 5

Figure S5. COSY NMR spectrum of compound **4** in CD_3OD 5

Figure S6. ^{13}C–DEPTQ NMR spectrum of compound **4** in CD_3OD at 151 MHz 6

Figure S7. Edited–HSQC NMR spectrum of compound **4** in CD_3OD 6

Figure S8. HMBC NMR spectrum of compound **4** in CD_3OD 7

Figure S9. ROESY NMR spectrum of compound **4** in CD_3OD 7

Figure S10. HRESIMS spectrum of compound **5** (negative ionization) 8

Figure S11. ^{1}H NMR spectrum of compound **5** in CD_3OD at 600 MHz 8

Figure S12. COSY NMR spectrum of compound **5** in CD_3OD 9

Figure S13. Edited–HSQC NMR spectrum of compound **5** in CD_3OD 9

Figure S14. HMBC NMR spectrum of compound **5** in CD_3OD 10

Figure S15. ROESY NMR spectrum of compound **5** in CD_3OD 10

Figure S16. HRESIMS spectrum of compound **6** (negative ionization) 11

Figure S17. ^{1}H NMR spectrum of compound **6** in CD_3OD at 600 MHz 11

Figure S18. COSY NMR spectrum of compound **6** in CD_3OD 12

Figure S19. Edited–HSQC NMR spectrum of compound **6** in CD_3OD 12

Figure S20. HMBC NMR spectrum of compound **6** in CD_3OD 13

Figure S21. ROESY NMR spectrum of compound **6** in CD_3OD 13

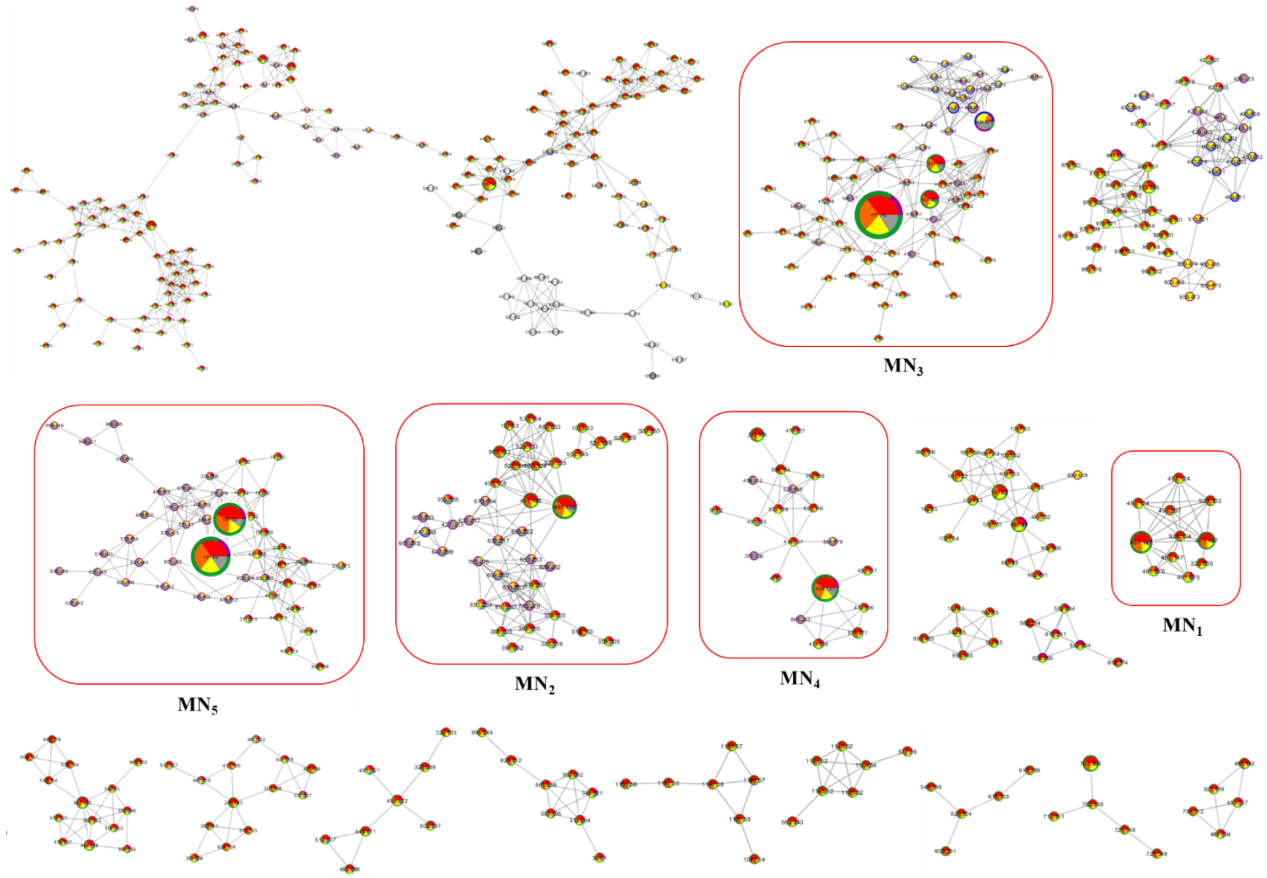

Figure S1. Twenty candidate bioactive clusters with a minimum of five nodes observed by visual inspection based on dominant red color tag and five selected bioactive clusters in red square box (MN$_1$–MN$_5$) were nominated based on node size.

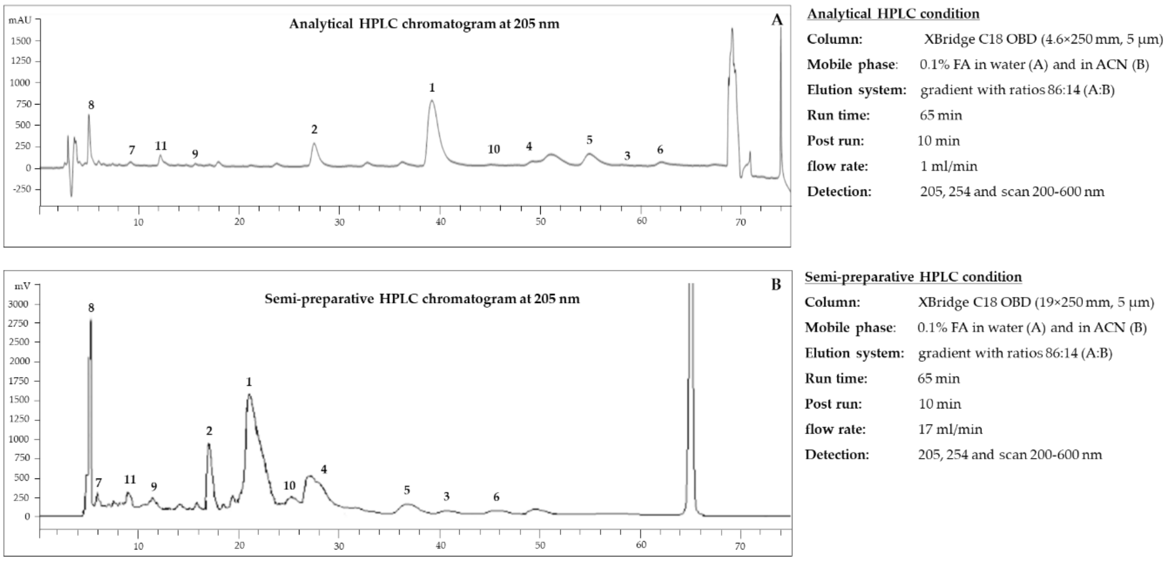

Figure S2. Representative HPLC chromatograms from method transfer between HPLC (**A**) and semi-preparative HPLC (**B**) for separation of compounds **1**–**11** in fraction 3 of MPLC of *B. monnieri* extract

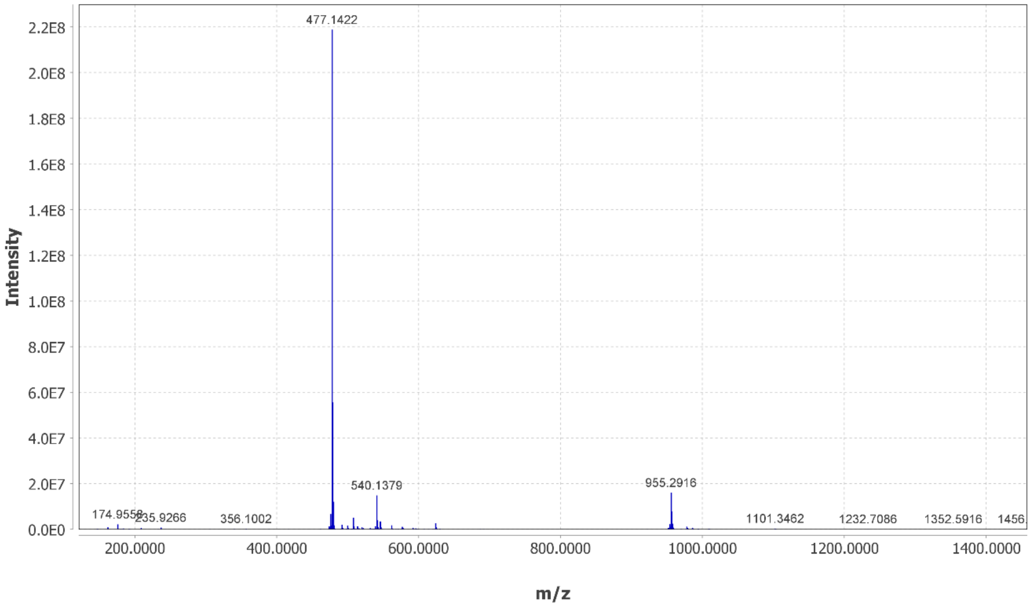

Figure S3. HRESIMS spectrum of compound **4** (negative ionization)

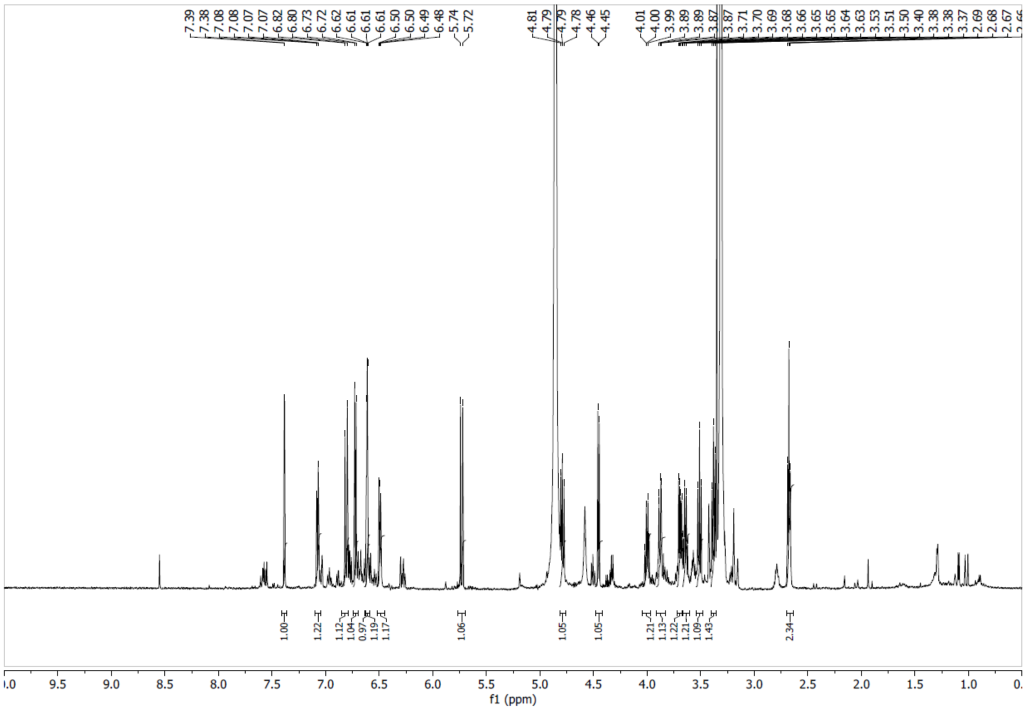

Figure S4. ^{1}H NMR spectrum of compound **4** in CD_3OD at 600 MHz

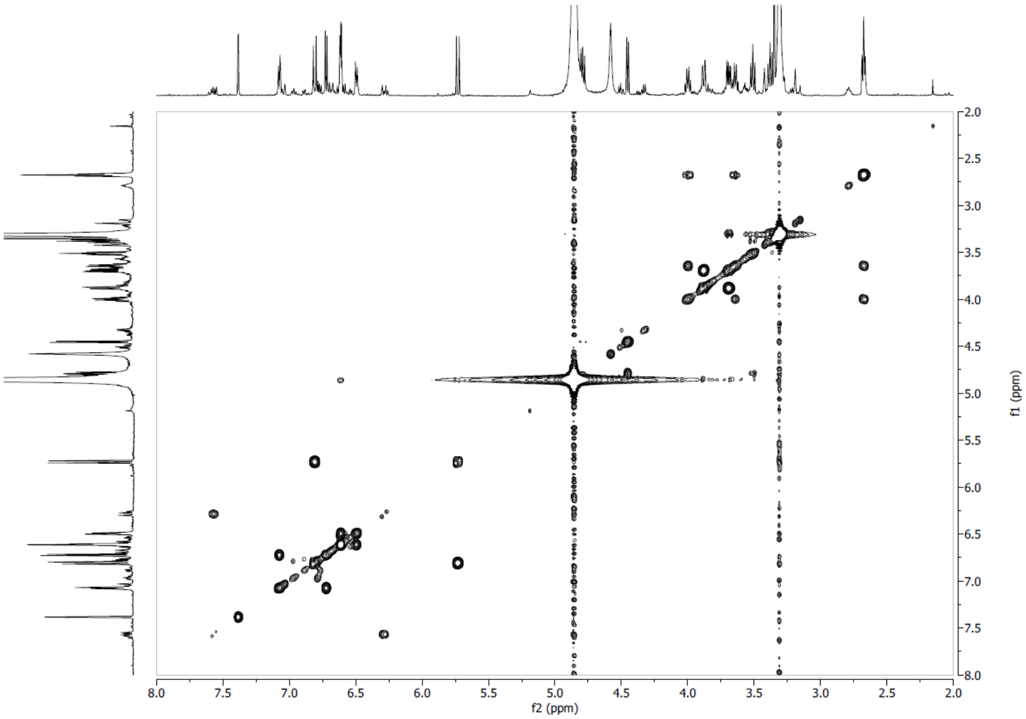

Figure S5. COSY NMR spectrum of compound **4** in CD_3OD

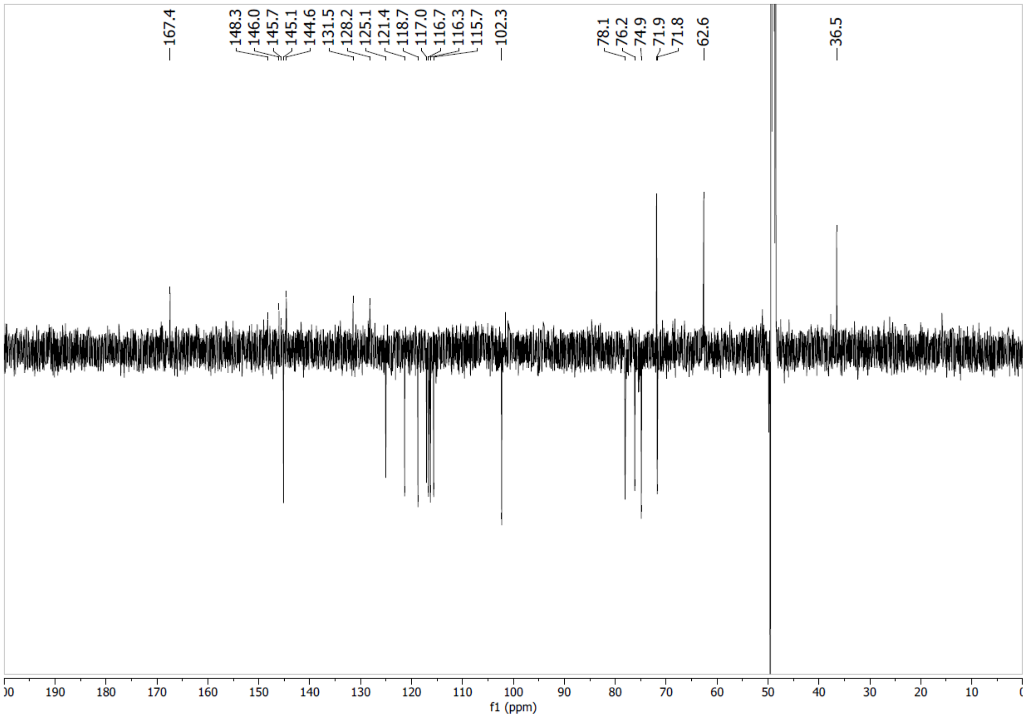

Figure S6. ^{13}C–DEPTQ NMR spectrum of compound **4** in CD_3OD at 151 MHz

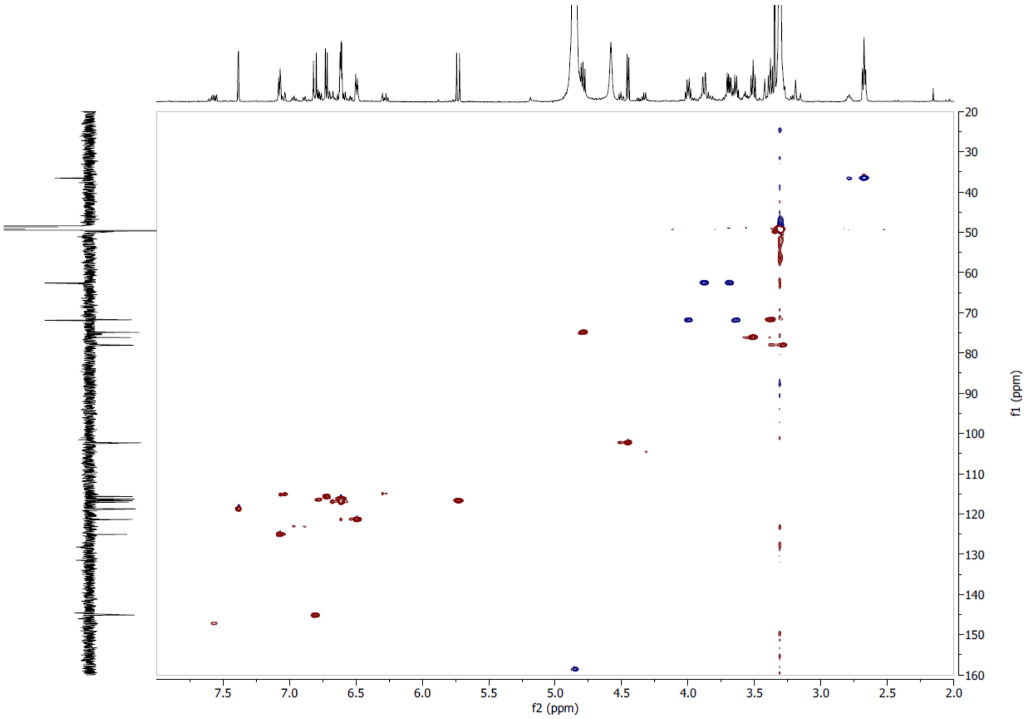

Figure S7. Edited–HSQC NMR spectrum of compound **4** in CD_3OD

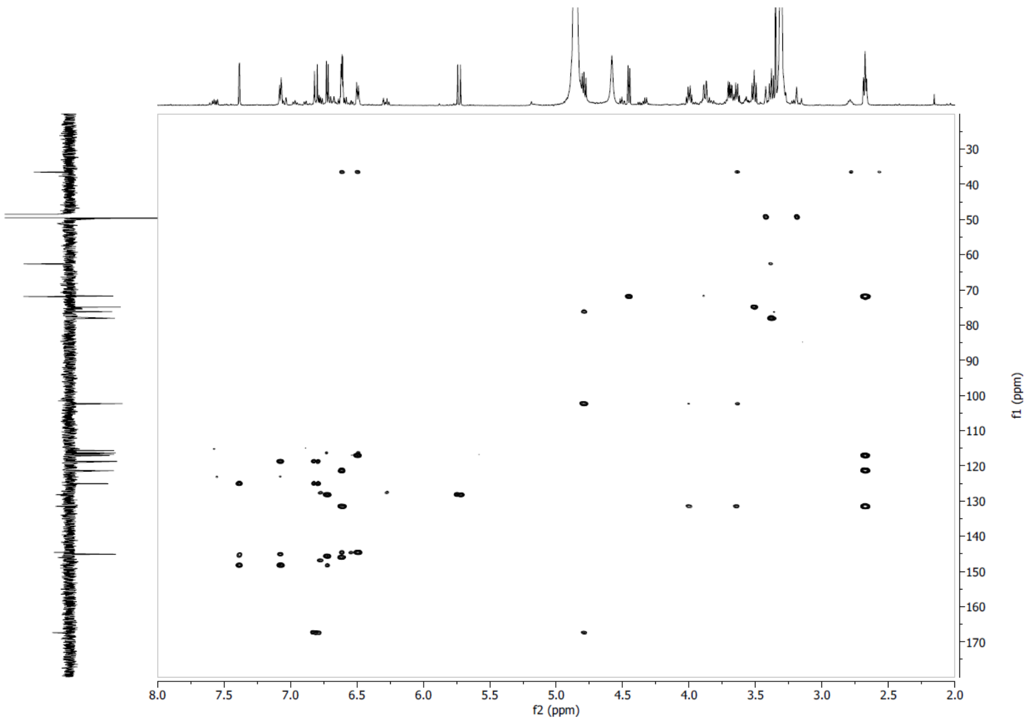

Figure S8. HMBC NMR spectrum of compound **4** in CD_3OD

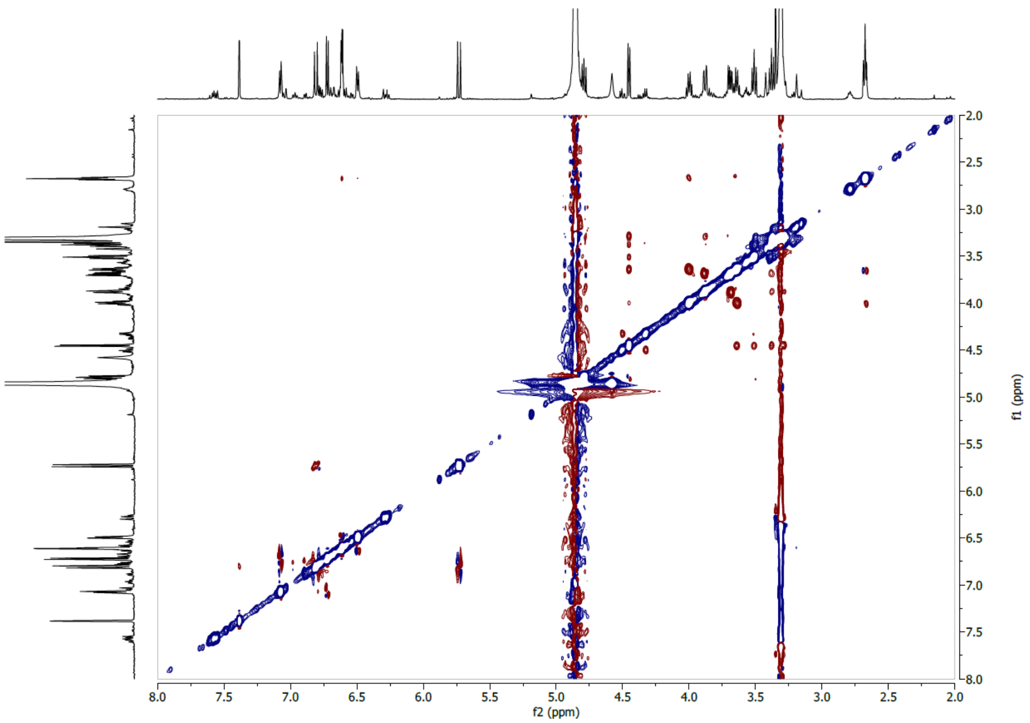

Figure S9. ROESY NMR spectrum of compound **4** in CD_3OD

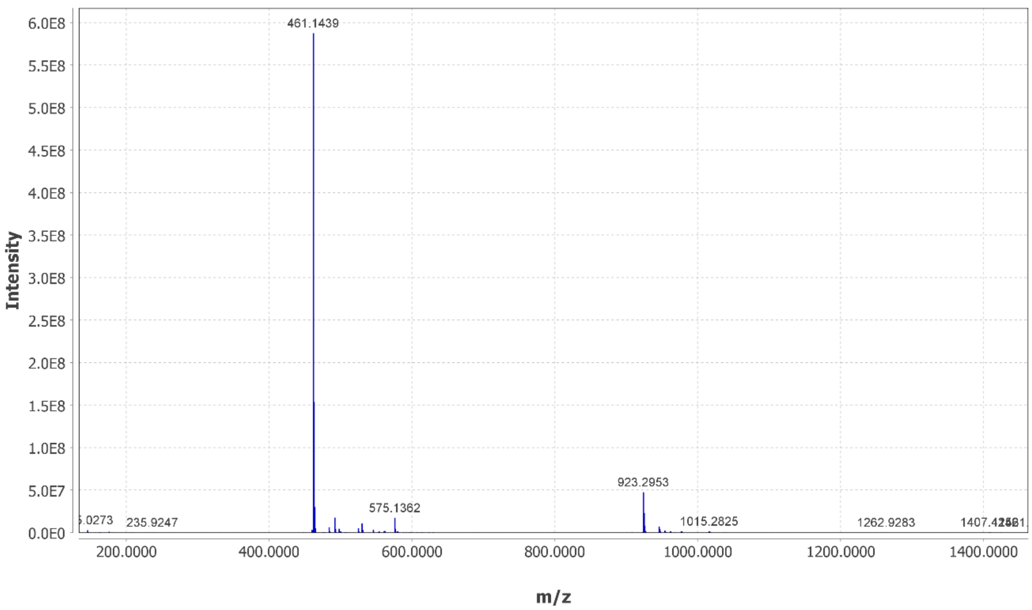

Figure S10. HRESIMS spectrum of compound **5** (negative ionization)

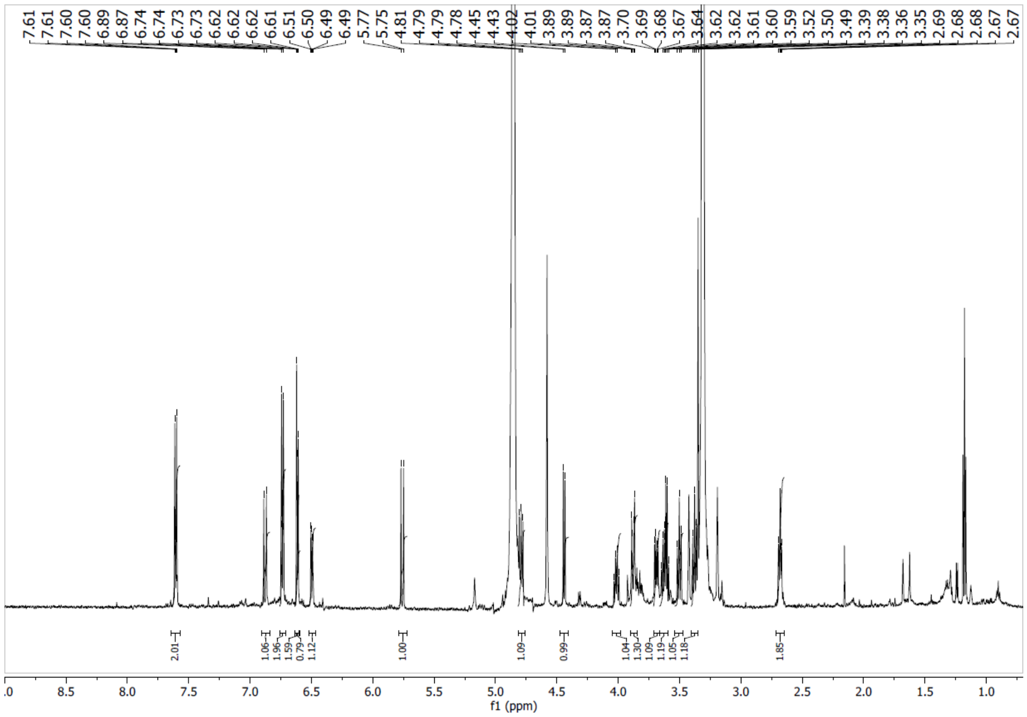

Figure S11. ^{1}H NMR spectrum of compound **5** in CD_3OD at 600 MHz

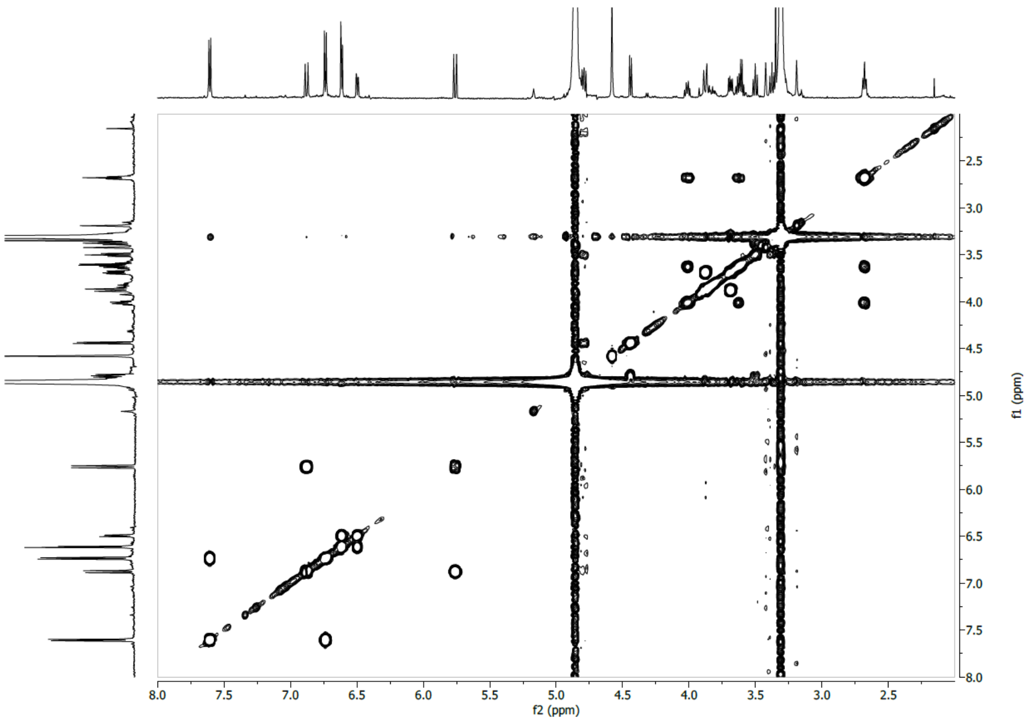

Figure S12. COSY NMR spectrum of compound **5** in CD_3OD

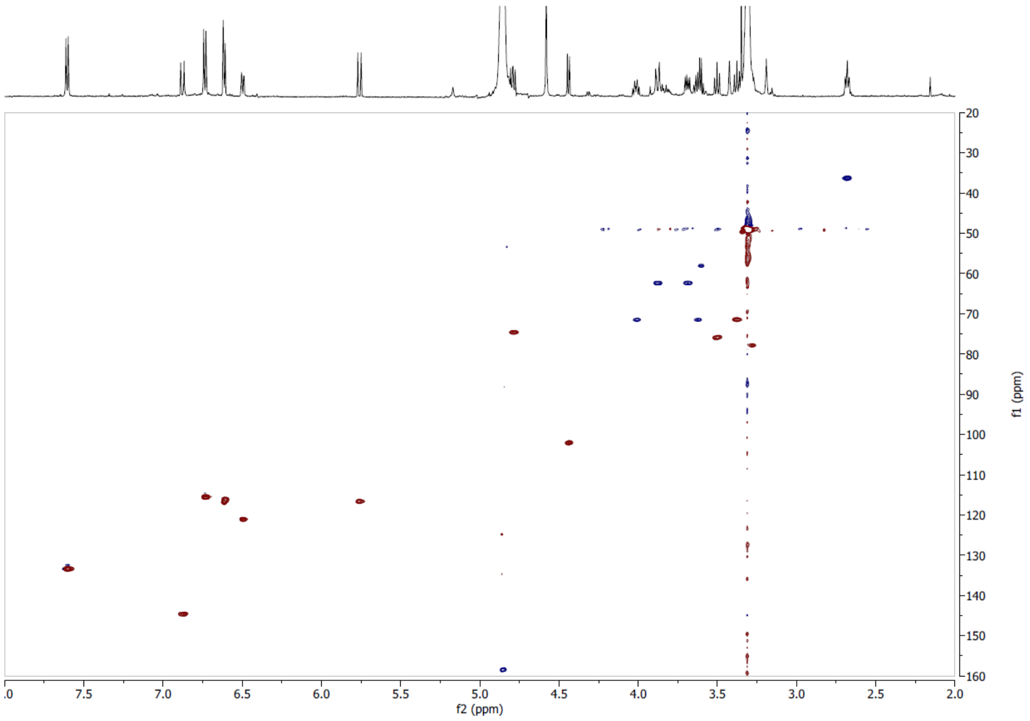

Figure S13. Edited–HSQC NMR spectrum of compound **5** in CD_3OD

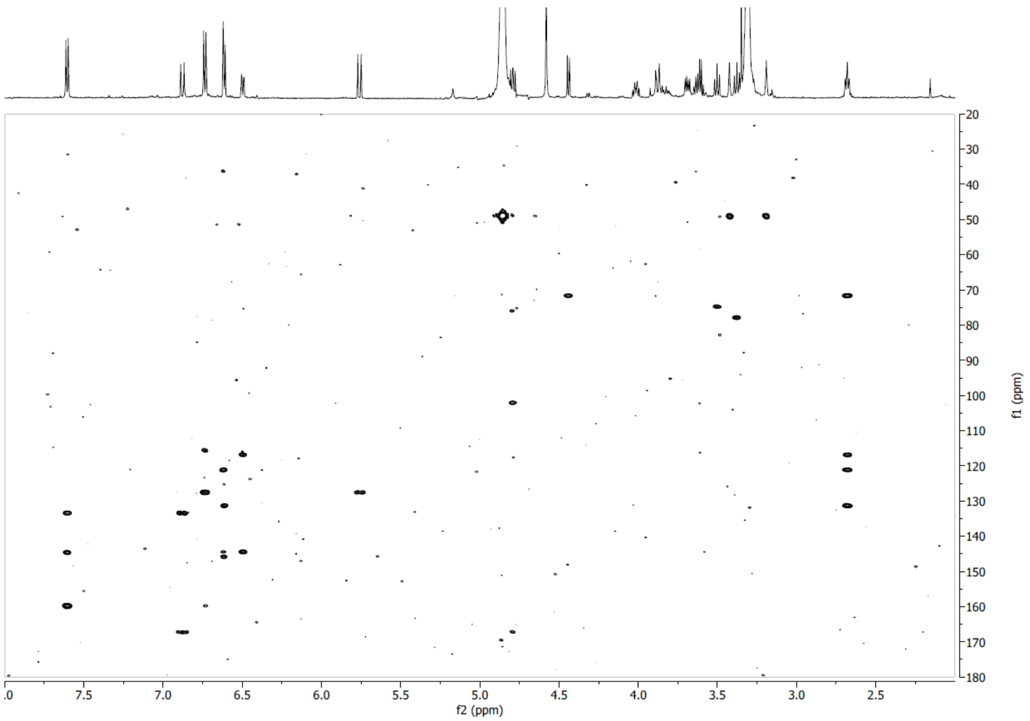

Figure S14. HMBC NMR spectrum of compound **5** in CD_3OD

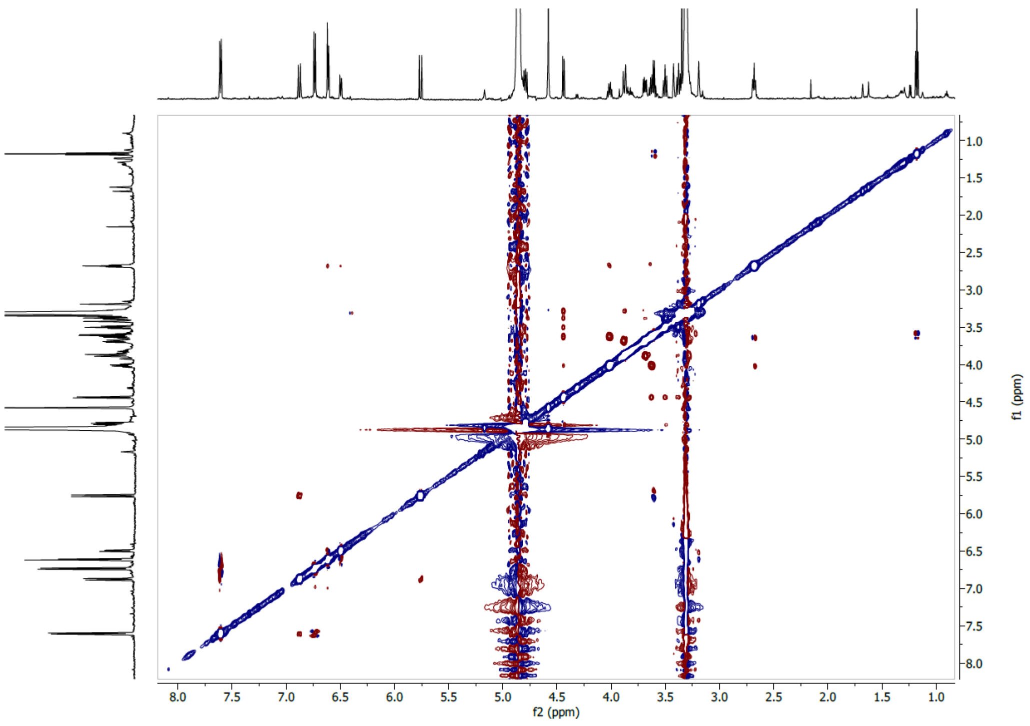

Figure S15. ROESY NMR spectrum of compound **5** in CD_3OD

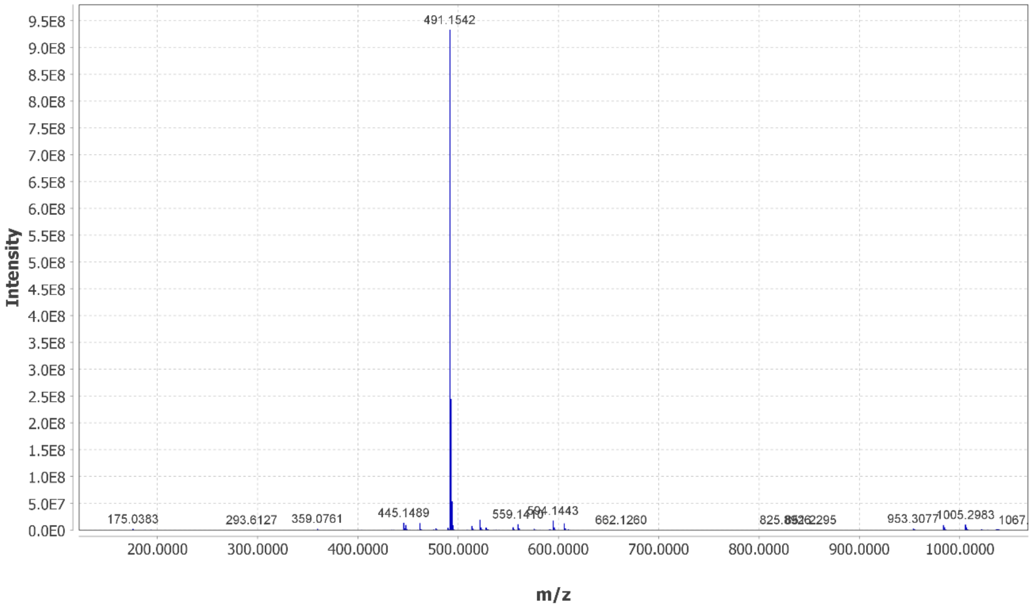

Figure S16. HRESIMS spectrum of compound **6** (negative ionization)

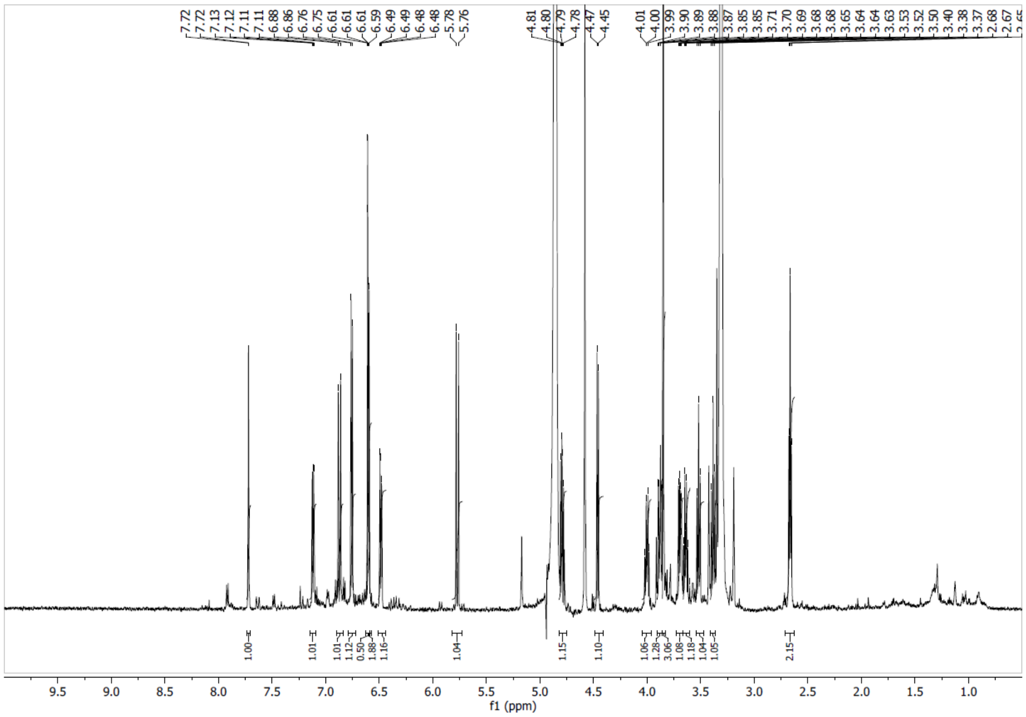

Figure S17. ^{1}H NMR spectrum of compound **6** in CD_3OD at 600 MHz

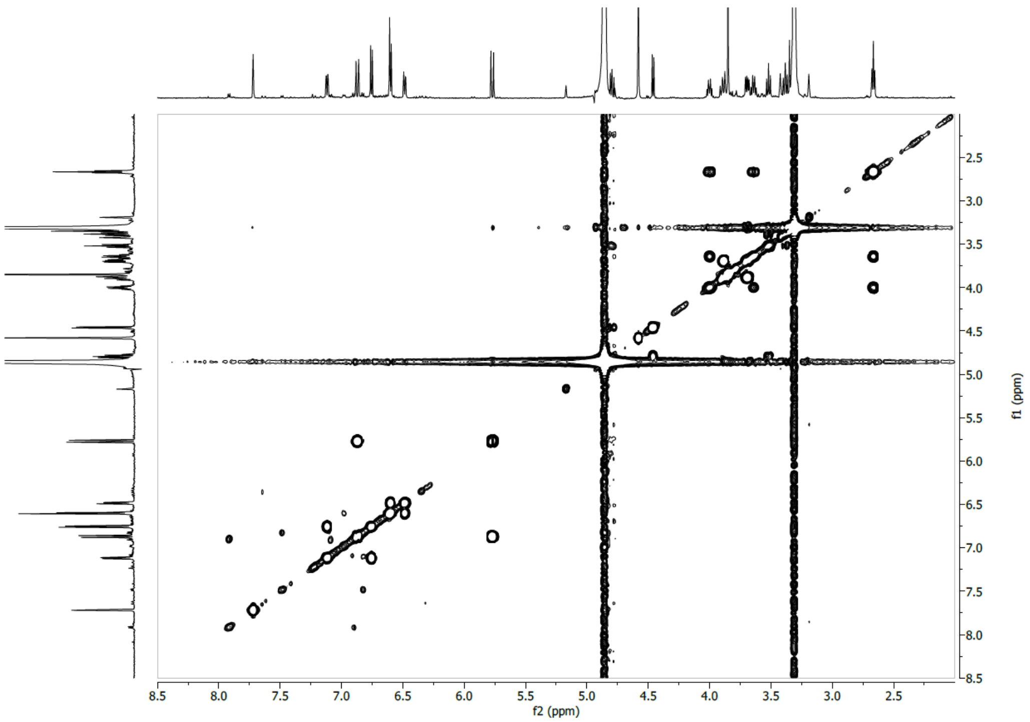

Figure S18. COSY NMR spectrum of compound **6** in CD_3OD

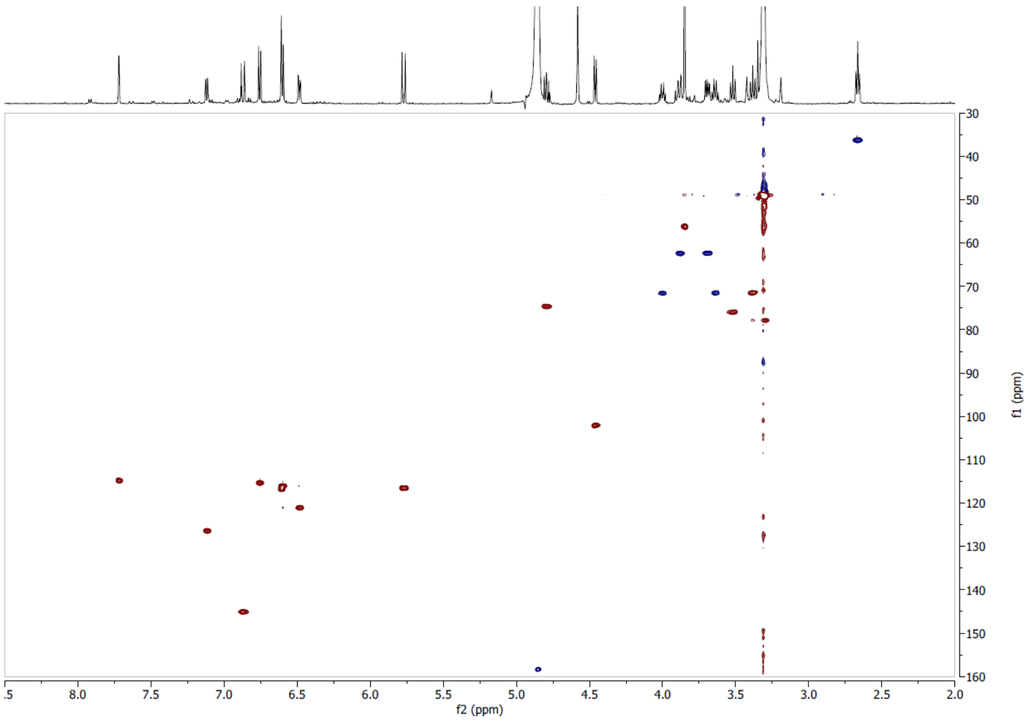

Figure S19. Edited–HSQC NMR spectrum of compound **6** in CD_3OD

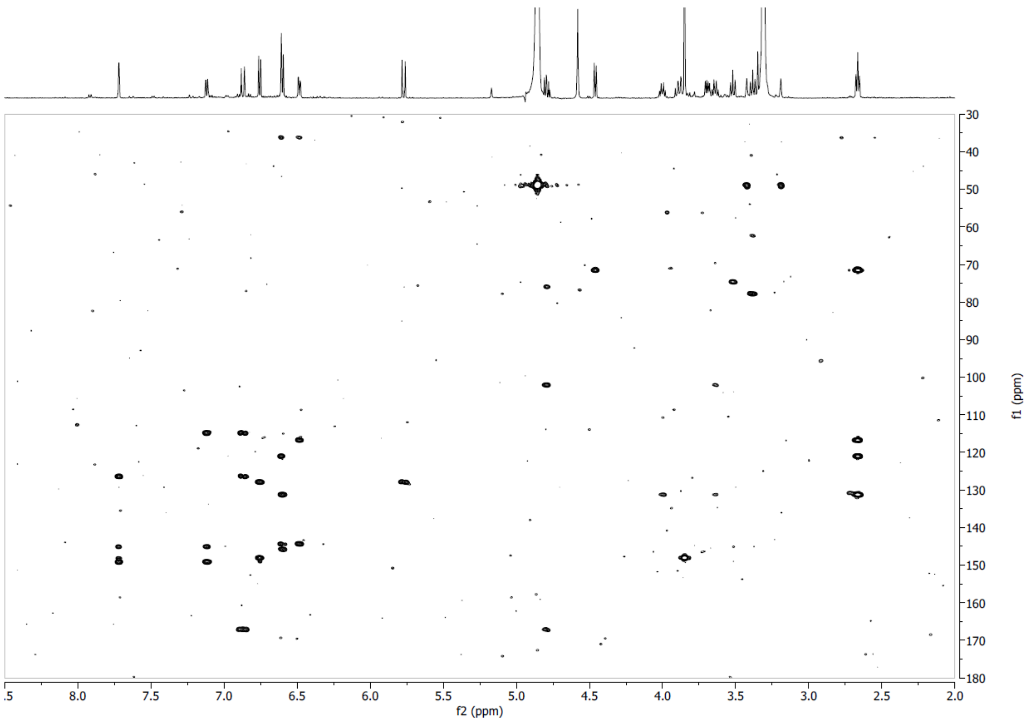

Figure S20. HMBC NMR spectrum of compound **6** in CD_3OD

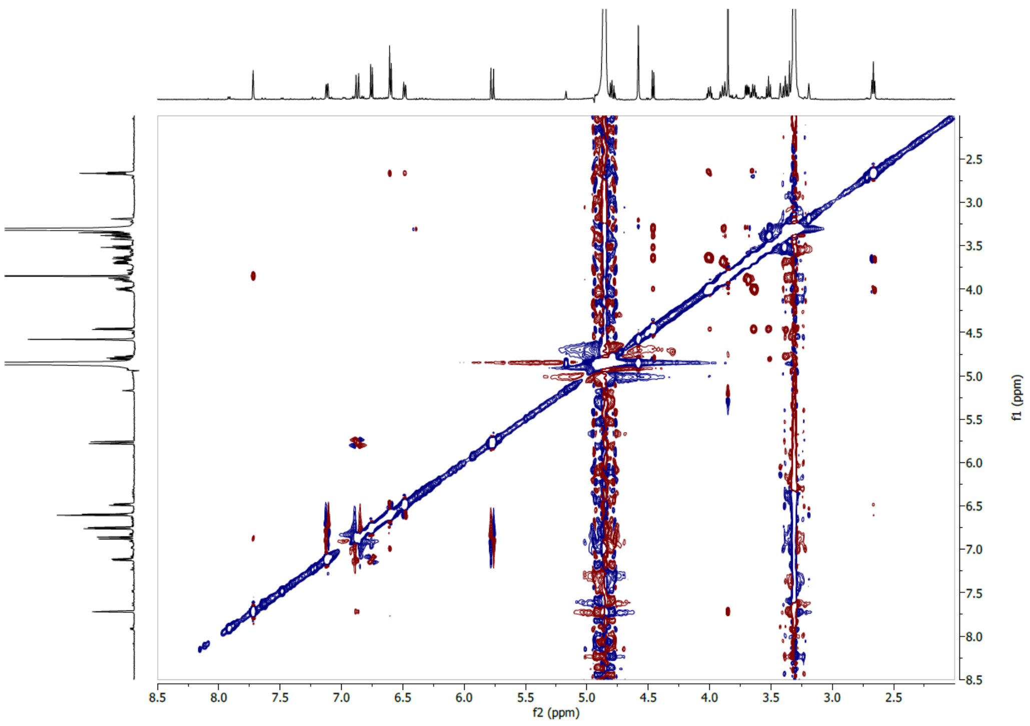

Figure S21. ROESY NMR spectrum of compound **6** in CD_3OD
